# Divergent allocation of sperm and the seminal proteome along a competition gradient in *Drosophila melanogaster*

**DOI:** 10.1073/pnas.1906149116

**Published:** 2019-08-20

**Authors:** Ben R. Hopkins, Irem Sepil, Marie-Laëtitia Thézénas, James F. Craig, Thomas Miller, Philip D. Charles, Roman Fischer, Benedikt M. Kessler, Amanda Bretman, Tommaso Pizzari, Stuart Wigby

**Affiliations:** ^a^Edward Grey Institute, Department of Zoology, University of Oxford, OX1 3PS Oxford, United Kingdom;; ^b^TDI Mass Spectrometry Laboratory, Target Discovery Institute, Nuffield Department of Medicine, University of Oxford, OX3 7BN Oxford, United Kingdom;; ^c^School of Biology, Faculty of Biological Sciences, University of Leeds, LS2 9JT Leeds, United Kingdom

**Keywords:** reproduction, sperm competition, seminal fluid, sexual selection, phenotypic plasticity

## Abstract

Sperm competition favors large, costly ejaculates, and theory predicts the evolution of allocation strategies that enable males to plastically tailor ejaculate expenditure to sperm competition threat. While greater sperm transfer in response to a perceived increase in the risk of sperm competition is well-supported, we have a poor understanding of whether males (*i*) respond to changes in perceived intensity of sperm competition, (*ii*) use the same allocation rules for sperm and seminal fluid, and (*iii*) experience changes in current and future reproductive performance as a result of ejaculate compositional changes. Combining quantitative proteomics with fluorescent sperm labeling, we show that *Drosophila melanogaster* males exercise independent control over the transfer of sperm and seminal fluid proteins (SFPs) under different levels of male–male competition. While sperm transfer peaks at low competition, consistent with some theoretical predictions based on sperm competition intensity, the abundance of transferred SFPs generally increases at high competition levels. However, we find that clusters of SFPs vary in the directionality and sensitivity of their response to competition, promoting compositional change in seminal fluid. By tracking the degree of decline in male mating probability and offspring production across successive matings, we provide evidence that ejaculate compositional change represents an adaptive response to current sperm competition, but one that comes at a cost to future mating performance. Our work reveals a previously unknown divergence in ejaculate component allocation rules, exposes downstream costs of elevated ejaculate investment, and ultimately suggests a central role for ejaculate compositional plasticity in sexual selection.

## Significance

Ejaculate quality plays an essential role in fertility, sperm competition, and offspring health. A key modulator of ejaculate quality is the social environment. Although males across taxa are known to strategically allocate sperm in response to rivals, how this applies to myriad other ejaculate components is poorly resolved. Here, we take a multilevel approach, from protein to fitness, to show that *Drosophila melanogaster* males divergently allocate sperm and seminal fluid proteins along a competition gradient. Using a combination of fluorescence-labeled sperm, quantitative proteomics, and multimating assays, we demonstrate that males are remarkably sensitive to the intensity of competition they perceive, show compositional change across and within portions of the ejaculate, and that this compositional change carries distinct costs and benefits.

The costs of producing an ejaculate were long thought to be trivial (1). It was therefore assumed that constraints on mate acquisition and female reproduction represented the principal limit on male reproductive potential (2). However, we now know that (*i*) males become depleted of sperm and seminal fluid through repeated mating (3–5), (*ii*) depleted ejaculates are associated with reduced fertilization success, particularly where sperm from different males compete for fertilizations (“sperm competition”) (6), and (*iii*) replenishing lost ejaculate material may be impossible, as in prospermatogenic species (7), or require considerable time and energy (4, 8, 9). The framework of “ejaculate economics” posits that these costs of depletion and replenishment shape how ejaculates are composed, produced, and transferred (10). Under this framework, males are predicted to be prudent when allocating ejaculate products, tailoring the quantity transferred to the threat of sperm competition (5).

Theory predicts that ejaculate expenditure strategies are modulated by the level of sperm competition, information, and patterns of sperm precedence (10). All else being equal, in populations where females vary in the probability of mating with more than one male, males are predicted to increase sperm allocation when they perceive a higher risk of sperm competition, which is defined as the probability of female double mating (11, 12). This prediction has been upheld in many animal groups, including birds, crustaceans, fish, insects, and mammals (13). When females always mate with multiple males, however, individual males are expected to respond to the number of competitors (“sperm competition intensity”) associated with a mating opportunity. Early theory showed that when males have perfect information regarding the number of competitors associated with the current opportunity, peak sperm transfer should occur in the presence of a single rival, declining under increasingly competitive conditions as the benefit of transferring more sperm decreases (14). But empirical support for these predictions is limited (13). More recent theory has argued that optimal allocation rules may be modulated by additional parameters such as the degree of fairness in the use of sperm from different males, female remating behavior, and tradeoffs between ejaculate expenditure and investment in other reproductive traits, such as mate searching (15–17).

A major shortcoming in the study of ejaculate expenditure has been to treat the ejaculate as a homogeneous entity. Rather, ejaculates are composites of many elements. Alongside sperm, the seminal fluid typically contains lipids, nucleic acids, extracellular vesicles, and proteins (seminal fluid proteins; SFPs) (18, 19). Across diverse taxa, these nonsperm seminal products variously act as crucial mediators of reproductive performance, female behavior, and even offspring phenotype (20–22). SFPs further play important roles in defending first-male paternity in competitive mating contexts. In *Drosophila melanogaster* and other insects, this is achieved in part by influencing rates of female oviposition and sperm use, and markedly reducing female receptivity to remating (20). Whether seminal fluid should follow the same allocation rules as sperm is unclear, but there is good reason to suspect not. Seminal fluid and sperm can deplete at different rates, such as in the bedbug *Cimex lectularius*, where seminal fluid availability, rather than sperm, ultimately constrains male mating (3). Moreover, the functions of seminal fluid constituents can select for novel strategies of ejaculate allocation that cannot apply to sperm. For example, the fecundity-enhancing effect of seminal fluid substances transferred by one male can be parasitized by other males subsequently mating with the same female (23). Consequently, some theory has shown that males can gain from independently modulating sperm and SFP transfer in relation to sperm competition risk when the fitness-enhancing effects of SFPs disproportionately benefit a female’s future partners (24).

Where attempts have been made to describe seminal fluid allocation patterns, two broad sets of limitations have been encountered. The first relates to characterizing seminal fluid change. A common approach is to investigate gene expression changes in SFP-producing tissues. While such studies have revealed an effect of the social environment in some insects (25–27), molluscs (28), and flatworms (29), they assume that changes in SFP gene expression correspond to changes in SFP transfer to females. This assumption is undermined by extensive evidence that ready-produced sperm and SFPs can be differentially transferred across matings (30, 31). Perhaps more significantly, gene expression studies rely on the regularly breached assumption that a difference in gene expression necessitates a difference in protein abundance (32, 33). An additional drawback is the focus on just a handful of SFPs (see also ref. 34). Insect, avian, and mammalian seminal fluid proteomes are known to be diverse, containing hundreds or even thousands of different proteins (35–37). Whether SFPs should respond uniformly is unclear, but gene expression and proteomics studies of insect, mollusc, flatworm, and mammalian seminal fluid-contributing tissues suggest that different proteins may respond differently to competition (25, 28, 29, 38).

The second set of limitations relates to characterizing the costs and benefits of ejaculate compositional change. Compositional changes that follow male exposure to perceived sperm competition have been associated with increased paternity shares and offspring production in the fruit fly *D. melanogaster* (39, 40). In this species, two functionally important SFPs have been shown to be transferred in greater quantities following male exposure to competition (34). The broad compositional changes that underlie these benefits, however, including potential interactions between sperm and SFP transfer, remain unidentified. Moreover, at the heart of the ejaculate economics framework is the idea that greater investment in one mating should come at the expense of those in the future. Whether these costs map onto allocation of distinct ejaculate components is unclear. Ultimately, attributing costs and benefits to different parts of the ejaculate is key to understanding the evolutionary significance of ejaculate expenditure plasticity (41, 42).

Here, we perform an integrated test of the allocation of ejaculate components in *D. melanogaster* after multiday, precopulatory exposure to different levels of competition from rivals: none (males held alone), low (males held in single-sex pairs), or high (males held in single-sex groups of 8). This approach mirrors the design previously used to show that male *D. melanogaster* exposed to rivals mate for longer (43), increase their paternity share (39), and show broad transcriptomic responses in sensory genes (44). More generally, this paradigm is regularly adopted when testing plastic responses to rivals (reviewed in ref. 45). We first use fluorescence labeling to test whether males change the number of sperm they transfer in response to competition. Next, we ask the same question of the seminal fluid proteome, applying label-free quantitative proteomics to virgin and postmating accessory glands, the primary production site of the ∼200 SFPs known to be transferred to females (46, 47). This approach simultaneously captures change in the production, transfer, and degree of depletion of SFPs, and provides a deep analysis of the seminal proteome (46). Finally, we test whether patterns of competition-dependent ejaculate compositional change affect indices of current and future reproductive success.

## Results and Discussion

### Sperm Transfer Peaks at Low Competition.

**Fig. 1.**
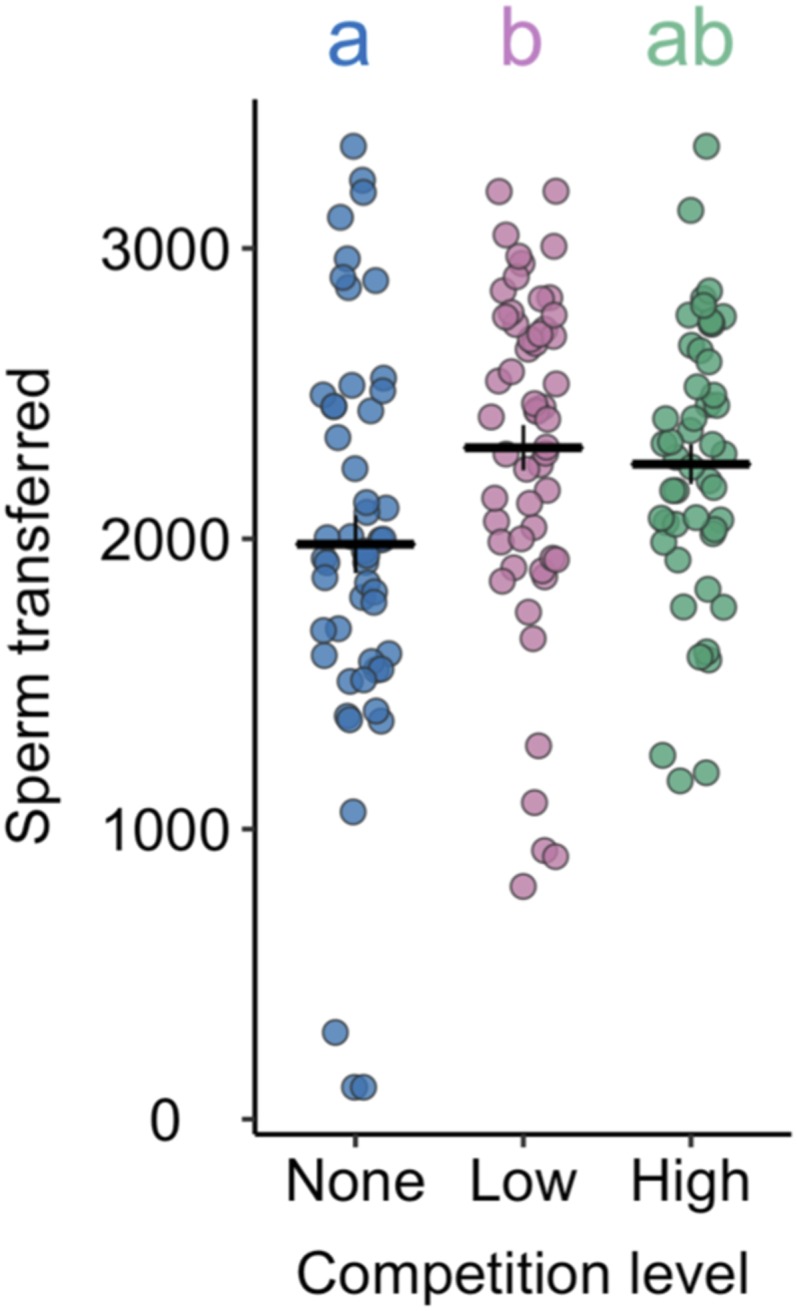
Males increase sperm transfer when exposed to rivals. Sperm counts from across all regions of the reproductive tract (bursa, seminal receptacle, spermathecae) of females frozen 25 min after the start of mating. Males were previously held alone (no competition), in a same-sex pair (low competition), or in a same-sex group of 8 (high competition). Lines give treatment mean with ±1 SE, *n*_no_ = 51, *n*_low_ = 54, *n*_high_ = 48, pooled from two replicates. Letters give significant comparisons at the *P* < 0.05 level.

We first counted fluorescently labeled sperm in the reproductive tract of newly mated females. We detected a significant effect of competition on sperm transfer (*F*_2,149_ = 3.43, *P* = 0.035; Fig. 1): Males exposed to low competition transferred an average of 333 more sperm than males exposed to no competition (*t* = 2.57, *P* = 0.011). This equates to an ∼17% increase, similar to the ∼20% increase reported when a *D. melanogaster* male is suddenly exposed to two rivals during mating (48). This increase in sperm transfer is likely facilitated by differences in sperm production: Previous work has shown that prolonged, precopulatory exposure to a single rival leads to elevated sperm production (49). We detected no significant difference in the number of sperm transferred by low- and high-competition males (difference of 57 sperm, *t* = 0.79, *P* = 0.433), and no significant difference between no- and high-competition males (difference of 276 sperm, *t* = 1.72, *P* = 0.087). Thus, sperm transfer peaks at low competition before showing a slight decline at higher levels of competition, as predicted by some theoretical models (14).

### Seminal Proteome Production and Transfer Peak at High Competition.

**Fig. 2.**
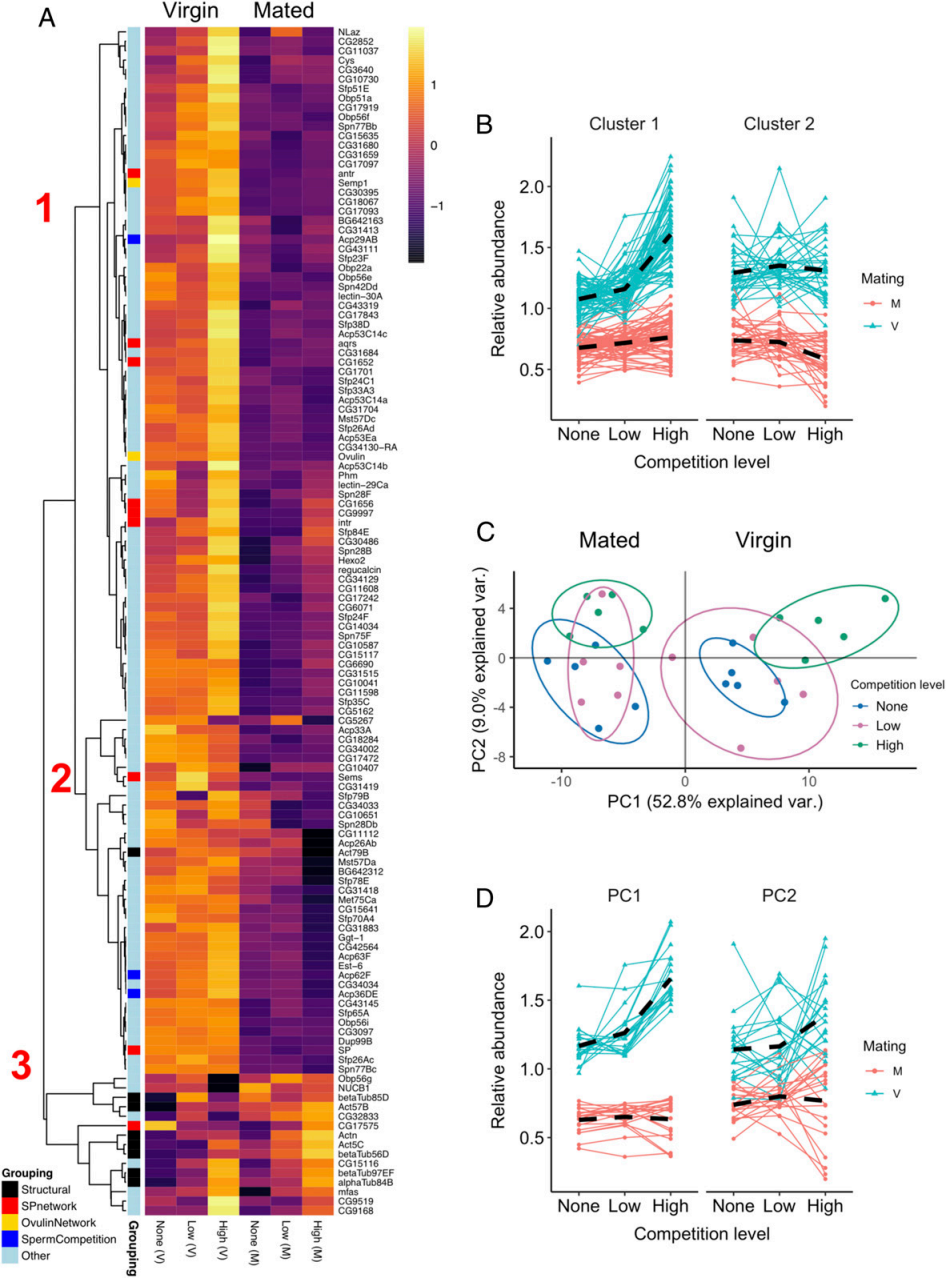
Seminal fluid protein production, transfer, and composition respond to competition intensity. Males were held alone (no competition), in a same-sex pair (low competition), or in a same-sex group of 8 (high competition) before being mated or retained as virgins. (*A*) Heatmap showing the abundance of 119 detected SFPs and 8 structural proteins detected in accessory glands. Each cell gives the across-replicate mean for that protein in a given treatment combination. From left to right, columns 1 to 3, virgins (V); columns 4 to 6, mated (M). Row annotations provide functional information relating to whether a protein is structural, functions as part of the sex peptide or ovulin networks, or has another known role in sperm competition (54–59, 94). Pearson correlation was used as the distance metric for the hierarchical clustering. Red numbers denote higher-order clusters referenced in the main text. (*B*) Abundance patterns of SFPs in the two major clusters identified in *A*. Each SFP is plotted separately in virgin (blue) and mated (red) glands. The dashed black lines give the mean abundance pattern across SFPs. Abundance values are relativized by means centering and averaging across replicates. (*C*) PCA biplot of detected SFPs. Points represent each of 30 samples colored according to competition treatment. Ellipses denote 80% normal probability. (*D*) Abundance pattern of the top 20 contributing SFPs to PC1 (*Left*) and PC2 (*Right*) determined in the PCA referred to in *C* and plotted following the approach in *B*.

Next, we applied quantitative proteomics to virgin and recently mated accessory glands to test patterns of SFP allocation in response to competition. We detected 1,277 proteins, and focused on the 119 of those known to be transferred to females (*Materials and Methods*). We performed a hierarchical clustering analysis to identify distinct patterns of abundance change within the SFP proteome in relation to both mating (i.e., whether a male was experimentally mated or retained as a virgin) and the level of competition. We included a group of 8 structural proteins to act as a control outgroup that should not change in abundance with mating. Three distinct higher-order clusters were identified (Fig. 2*A*). Cluster 3 was highly enriched for the outgroup control—the structural proteins—and so was omitted from further analysis.

In clusters 1 and 2, the overall degree of difference in protein abundance between virgin and mated glands, which represents the quantity lost during mating, varied significantly with the level of competition (mating x competition: cluster 1: *F*_2,350_ = 36.32, *P* < 0.0001; cluster 2: *F*_2,170_ = 7.23, *P* = 0.001; Fig. 2*B*). However, the clusters reveal split responses to competition within the seminal proteome. Cluster 1 accounts for the majority of SFPs (74/119 SFPs) and captures a general pattern of marked increase in protein abundance in virgin high-competition glands (Fig. 2*B* and *SI Appendix*, Table S1). The more limited difference between competition treatments in mated glands suggests that greater production of SFPs in cluster 1 generally translates into greater transfer to females. Males across competition treatments are therefore left similarly SFP-depleted after mating. In contrast, SFPs in the smaller cluster 2 (38/119 SFPs) give a mixed response to competition, showing either no change or lower abundance at high competition. The overall trend in this cluster is for no difference in abundance in virgin glands but significantly greater postmating depletion in high-competition glands (Fig. 2*B* and *SI Appendix*, Table S1). Thus, for some SFPs it is the proportion that is transferred, rather than just the amount produced, that changes with the level of competition, a pattern hinted at in a previous study (31).

### The Composition of the SFP Proteome Changes in Response to Level of Competition.

We next asked whether SFPs vary in their sensitivity to the level of competition to drive compositional change in seminal fluid. A PCA showed that relative to the no-competition treatment, the seminal fluid proteome shows distinct compositional change at high, but not low, competition (Fig. 2*C*). PC1 explained over half of the variation in the data (52.8%; eigenvalue = 62.9; *SI Appendix*, Table S2) and the extracted scores were significantly associated with an interaction between mating and level of competition (*F*_2,20_ = 4.94, *P* = 0.018; *SI Appendix*, Table S2). We suggest that this interaction captures change in the abundance of SFPs transferred to females in the ejaculate during mating. PC2, which explained 9.0% of the variance (eigenvalue = 10.7), represented an axis of variation significantly associated with the level of competition (*F*_2,22_ = 12.79, *P* = 0.0002; *SI Appendix*, Table S2). Visual inspection of the abundance patterns of the top 20 contributing proteins to each PC revealed elevation in SFP production and transfer at high competition in PC1 (Fig. 2*D*) alongside variation in the relative responsiveness, direction of change, and degree of postmating retention of SFPs in PC2 (Fig. 2*D*). While almost all of the top 20 contributing proteins to PC1 belong to cluster 1 in our hierarchical clustering analysis (cluster 1: 17; cluster 2: 3; cluster 3: 0; Fig. 2*A*), cluster membership in PC2 is more variable (cluster 1: 9; cluster 2: 8; cluster 3: 3; Fig. 2*A*). This is consistent with nonuniformity across the seminal proteome in the responses of SFPs to competition.

### Functionally Important Sperm Competition SFPs Show Competition-Specific Up-Regulation.

To identify SFPs showing high-confidence change in response to competition, we used a differential abundance analysis. Across all samples, we detected 45 SFPs that showed a significant response to the level of competition, 38 of which showed peak abundance in high-competition virgin glands (*SI Appendix*, Fig. S1 and Table S3). Curiously, the remaining 7 included some at lowest abundance in high-competition glands. Overall, this list of 45 differentially abundant SFPs showed no significant associations with gene ontology terms in a DAVID search (50, 51), suggesting that SFPs belonging to disparate functional classes are similarly changed in response to competition. This is consistent with seminal fluid’s activity depending on interactions between molecules from a rich variety of biochemical classes (52). We also sought to understand the regulatory differences that underlie between-SFP variation in sensitivity to competition. Recent work has shown that groups of SFPs share putative binding sites for particular miRNAs (53). Thus, it may be that specific miRNAs are responsible for driving the changes in SFP expression that facilitate strategic changes in ejaculate composition. We tested this by asking whether the degree of change in the quantity of a given SFP transferred to females depends on the identity of the miRNA that regulates it. However, we found no support for this idea (*SI Appendix*, Fig. S2 and Table S4).

Within the 45 differentially abundant SFPs, we found some that showed highly dynamic responses to the level of competition. Seven are twice as abundant in high-competition virgin glands compared with no- and/or low-competition virgin glands (*SI Appendix*, Table S3). Of these 7, functional information is only available for Acp29AB, which is known to enter into the female sperm storage organs (54) and has been linked to sperm competition performance in association studies (55, 56). The remaining 6 comprise a cysteine protease inhibitor (Cys), an oxidoreductase (CG9519), an alkaline phosphatase-like enzyme (CG9168), and three with no available molecular information (Acp53C14b, CG43111, and Sfp38D). CG43111 and CG9519 are newly discovered SFPs (46). Collectively, this cluster of 7 especially dynamic SFPs contains prime candidates for proteins that play key roles in determining the outcome of postcopulatory competition, perhaps through effects on sperm competitiveness or female sperm use.

Among these 45 differentially abundant SFPs, a further 6 are known “sex peptide network” proteins (antr, aqrs, intr, CG1652, CG1656, and CG9997), which are all at highest abundance in virgin high-competition glands. Each of these 6 contributes to the binding of sex peptide to sperm, a process required for the long-term persistence of reduced female receptivity to remating, effective sperm release, and fecundity stimulation (57–59). These phenotypes are known mediators of sperm competition outcome (60, 61). Of the 6 network proteins we found at significantly higher abundance at high competition, all but aqrs have been shown to bind to sperm and enter into the female sperm storage organs (62).

### Greater SFP Production in High-Competition Males Correlates with a Steeper Rate of Decline in Mating Probability.

**Fig. 3.**
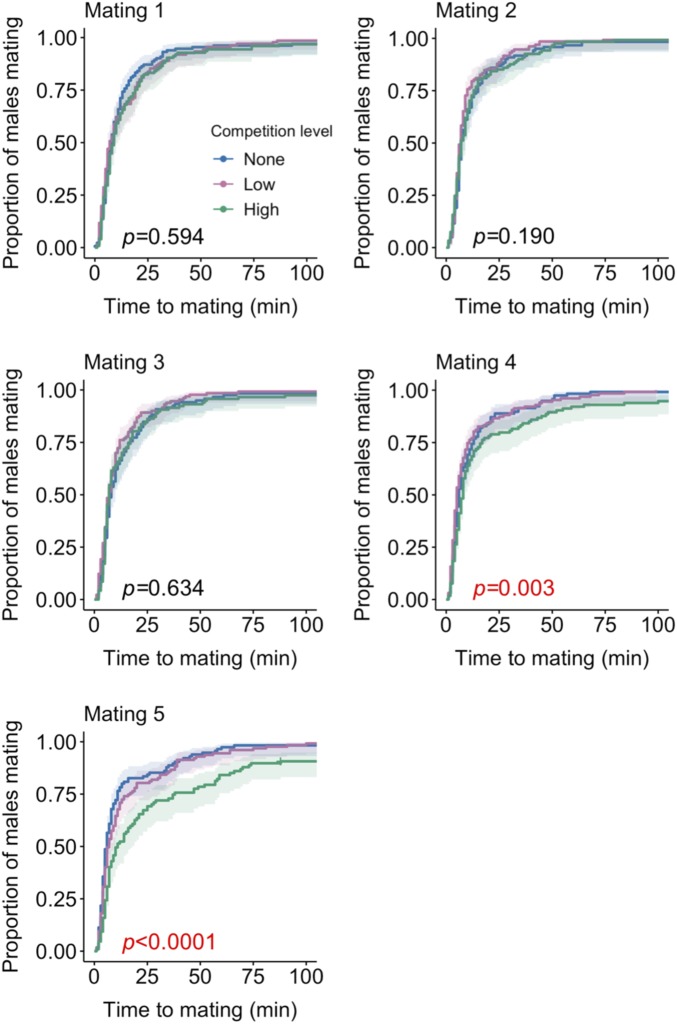
High-competition males show reduced mating completion after multiple matings. Males were held alone (no competition), in a same-sex pair (low competition), or in a same-sex group of 8 (high competition) before being provided with 5 successive virgin females. Male latency to mating is plotted for each mating. *P* values are for the overall effect of competition. Data were pooled from two replicates. Confidence intervals are given at 95%. At the start of first mating, *n*_no_ = 133, *n*_low_ = 137, *n*_high_ = 131. By the end of fifth mating, *n*_no_ = 121, *n*_low_ = 133, *n*_high_ = 114.

Our proteomics and sperm data suggest that males produce and transfer a different ejaculate composition at each of the competition levels we tested. To test whether these compositions covary with reproductive outcome and whether they come at a cost to future reproductive performance, we explored the rate of reproductive decline across 5 consecutive matings for males held alone (no competition), in a same-sex pair (low competition), or in a same-sex group of 8 (high competition). We first tested whether the level of competition influences the latency from a male’s first exposure to a female to the start of mating, which represents a proxy for the ability to acquire or compete for matings. We failed to find any difference in latency to mating between levels of competition over a male’s first 3 matings (mating 1: *LRT* = 1.04, *P* = 0.594; mating 2: *LRT* = 3.32, *P* = 0.190; mating 3: *LRT* = 0.226, *P* = 0.634; Fig. 3). However, by the fourth mating, high-competition males were significantly slower to mate and fewer ultimately did mate (*LRT* = 11.39, *P* = 0.003). This effect was larger again in the fifth mating (*LRT* = 22.23, *P* < 0.0001).

The reduction in the probability of mating in high-competition males may reflect males being in reduced condition owing to the increased density and/or male–male interactions in the precopulatory environment. Were this the case, then it may also be that the ejaculate allocation patterns we detect are driven by terminal investment-like mechanisms (63), rather than strategic responses to rivals. However, there is little evidence to suggest that competition-exposed males will differ in condition due to antagonistic interactions because male–male aggression is suppressed under the conditions imposed by the current experimental design, namely exposure to rivals was prolonged (64), females were absent (65), and food was abundant (66). While we cannot entirely rule out competition and density effects on male condition, the costs of protracted rival exposure are known to manifest late in life, after much longer periods of cohabitation. Indeed, when same-sex groups of males are held at even higher densities (12 males) than used in our high-competition treatment (8 males), there is no evidence of reduced reproductive performance after 21, compared with 7, days of continued exposure (67). Moreover, it takes over 35 d for males housed in single-sex pairs to show condition-related decline in reproductive performance and activity (40) and immunocompetence (68). Thus, it is more likely that the change in ejaculate investment following exposure to rivals represents a plastic strategic response to changes in the probability, and perhaps intensity, of postcopulatory competition, rather than a response to reduced male condition. Consequently, the reduced mating probability of high-competition males more likely reflects (*i*) males sensing that they are more seminal fluid-depleted, which may reduce their propensity to remate, and/or (*ii*) reduced capacity for mating due to a tradeoff resulting from higher investment in SFP production and transfer.

### Accessory Gland Replenishment Rate Is Unaffected by Exposure to Competition.

We next sought to test whether high-competition males are (*i*) more seminal fluid-depleted after 5 consecutive matings, and (*ii*) suffer consequences consistent with reduced condition. Repeated mating is known to reduce the size of the accessory glands, presumably through the emptying of the lumen and the expulsion of stored SFPs (69). If gland size reflects the quantity of stored seminal fluid, then we would predict high-competition males to show reduced gland size after repeated matings. Similarly, if high-competition males are in reduced condition, then we would predict that their accessory glands would refill with newly synthesized SFPs, and thus increase in size, at a reduced rate. To test this, we measured the size of accessory glands from males dissected at different time points after their 5 matings. We failed to find a significant effect of competition treatment in accessory gland size either directly (*F*_2,294_ = 1.36, *P* = 0.259; *SI Appendix*, Fig. S3) or through an interaction with time (*F*_2,292_ = 0.09, *P* = 0.919). However, we did find that gland size significantly increased with time since mating (*F*_1,294_ = 669.26, *P* < 0.0001). The normal rate of replenishment observed in high-competition males relative to the no- and low-competition treatments indicates either that increased SFP investment in response to high competition is cost-free, or that high-competition males compensate for such costs by withdrawing investment from other reproductive traits or somatic maintenance. Such a reallocation could come at the expense of precopulatory traits, which would be consistent with our finding that high-competition males show reduced mating probability (Fig. 3).

### Greater SFP Production in High-Competition Males Correlates with a Steeper Rate of Decline in Offspring Production.

**Fig. 4.**
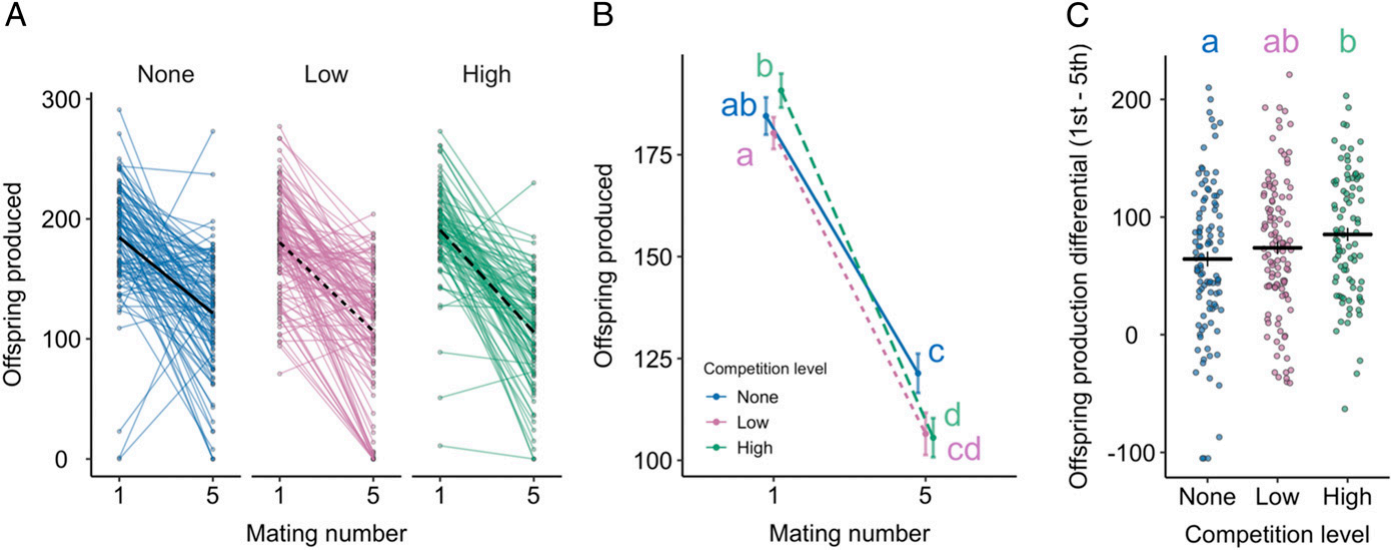
Male exposure to competition changes offspring production across matings. Males were held alone (no competition), in a same-sex pair (low competition), or in a same-sex group of 8 (high competition) before being provided with 5 successive virgin females. (*A*) Offspring produced over 3 d after a female mated to a male on his first or fifth mating plotted separately in relation to competition treatment. Lines connect matings from the same individual. Black lines give the mean response. (*B*) The mean ± SE for each of the competition treatments plotted in *A*. (*C*) The per-male difference in first- and fifth-mating 3-d offspring production. Positive values indicate that more offspring were produced following a male’s first mating. Mean ± SE is given. Data were pooled from two replicates. *n*_no_ = 103, *n*_low_ = 112, *n*_high_ = 89. Letters give significant comparisons at the *P* < 0.05 level.

Previous work in *D. melanogaster* has shown that females mated to competition-exposed males produce more offspring (39, 40), suggesting that males perceiving a higher level of sperm competition transfer a more potent fecundity-stimulating ejaculate (e.g., ref. 34). However, elevated investment in one mating may come at a cost to future reproductive performance. Consistent with this idea, we detected a significant interaction between competition level and whether a male was mating for the first or fifth time on the number of offspring that his mate produced over a 3-d period (*F*_2,301_ = 3.16, *P* = 0.044; Fig. 4 *A* and *B*). High-competition males produced more offspring in their first mating (mean ± SE; 191 ± 4) compared with both low- (180 ± 4; *t* = 2.21, *P* = 0.028) and no-competition (185 ± 5; *t* = 1.03, *P* = 0.302) males, albeit not significantly so in the latter. That the trends for sperm transfer were lower for high-competition males than for low-competition males (Fig. 1) indicates that sperm cannot explain the pattern of offspring production; instead, elevated SFP transfer (Fig. 2) likely explains the increased offspring production following the first mating in the high-competition treatment.

For the most part, matings with fifth-mating males produced offspring (i.e., were fertile), contrasting with a previous claim that *D. melanogaster* males are infertile after 3 or 4 consecutive matings (70). However, both low- and high-competition males performed relatively poorly in stimulating offspring production in the fifth mating compared with no-competition males. High-competition males produced on average 16 fewer offspring than no-competition males (∼13% reduction; *t* = 2.09, *P* = 0.037; Fig. 4 *A* and *B*). Low-competition males produced on average 15 fewer offspring than no-competition males, although this difference was not significant (∼12% reduction; *t* = 1.67, *P* = 0.096; Fig. 4 *A* and *B*). Competition-exposed males thus showed a stronger decline in offspring output between the first and fifth matings, which is consistent with them being more SFP-depleted compared with the no-competition males. However, our accessory gland size data suggest that this difference in SFP depletion is not reflected in differences in gland size after 5 matings (*SI Appendix*, Fig. S3). We offer two explanations for this. First, although males from across the three competition treatments end their five matings with glands of the same size, they are likely to have not started that way given the significantly elevated SFP abundances that we detect in high-competition males prior to their first mating. In which case, males from the different competition treatments are likely to have been transferring different quantities of SFPs across their previous matings. Second, it is unclear to what extent the size of the gland reflects its internal composition: Differences in size may fail to capture differences in water content, SFP proteome composition, and the quantity of low-abundance, but functionally important, SFPs.

To better understand the treatment-specific decline between the first and fifth matings, we calculated the difference in the number of offspring produced in each mating, for each male. This analysis revealed a significant effect of competition intensity (*F*_2,300_ = 3.03, *P* = 0.050; Fig. 4*C*). Pairwise comparisons showed the only significant difference was between no- and high-competition males (*t* = 2.46, *P* = 0.014; no vs. low: *t* = 1.16, *P* = 0.246; high vs. low: *t* = 1.35, *P* = 0.167), with high-competition males showing a greater between-mating difference (“offspring production differential”), consistent with a tradeoff between relatively higher first-mating and lower fifth-mating investment.

### Mating Duration Aligns Poorly with Ejaculate Compositional Change.

**Fig. 5.**
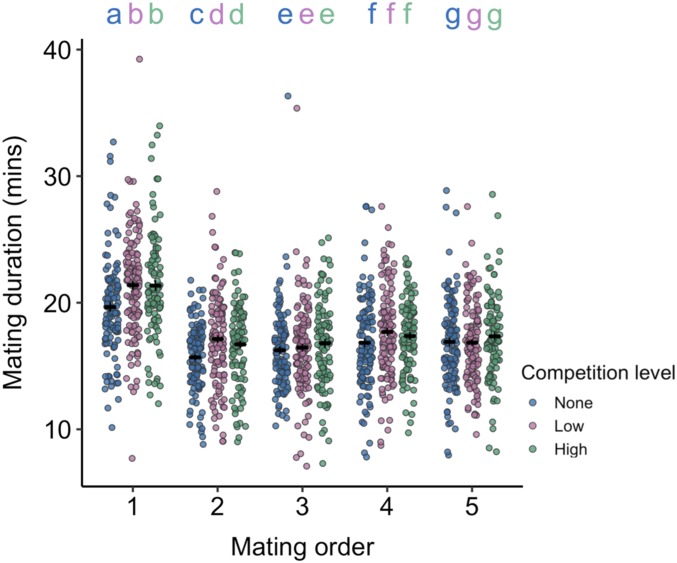
Males elevate mating duration in response to competition, but this effect is lost through repeated mating. Males were held alone (no competition), in a same-sex pair (low competition), or in a same-sex group of 8 (high competition) before mating to 5 successive virgin females. The duration of each mating in minutes is plotted, along with mean ± SE. Data were pooled from two replicates. The same flies are plotted for each mating. *n*_no_ = 113, *n*_low_ = 126, *n*_high_ = 100. Letters give significant pairwise comparisons within a mating number at the *P* < 0.05 level.

Mating duration is a widely used proxy for changes in ejaculate size, particularly in insects (reviewed in ref. 13). As in previous work (e.g., refs. 39 and 43), we find that males exposed to rivals (i.e., low and high competition) mate for longer than males exposed to no competition. However, we find a significant interaction between competition treatment and mating order, with an elevation in mating duration only persisting for the first of 2 consecutive matings and not for the subsequent 3 (competition x mating: *F*_8,1344_ = 2.03, *P* = 0.040; Fig. 5 and *SI Appendix*, Table S5). Male *D. melanogaster* have previously been shown to retain elevated mating durations if continuously exposed to rivals throughout their life and provided with restricted mating opportunities (40). However, our data suggest that competition-dependent elevation is not maintained across successive matings within a short time period. Exactly what elevated mating duration reflects is unclear, but a clear association with sperm or SFP transfer in *D. melanogaster* is doubtful: Sperm transfer and copulation duration are neuronally separable (71), greater sperm transfer can be associated with no change in duration (72) or even shorter matings (48), and the restriction of sperm transfer to a short window early on in a copulation complicates any relationship that may exist (73). Our data further suggest that differences in mating duration fail to capture changes in SFP transfer and the difference in offspring produced by low- and high-competition males.

### The Benefits of Elevated Ejaculate Investment Do Not Extend to All Postmating Phenotypes and Depend on Female Intermating Interval.

As part of our 5-mating experiment, we failed to find an effect of competition on either defensive sperm competition performance (competition x mating: *F*_2,175_ = 0.920, *P* = 0.400; competition, *F*_2,353_ = 0.105, *P* = 0.901; *SI Appendix*, Fig. S4*A*) or female receptivity to remating (competition x mating: *LRT* = 1.06, *P* = 0.590; competition, *LRT* = 0.28, *P* = 0.869; *SI Appendix*, Fig. S4*B*), although male performance in both these traits was significantly diminished after multiple matings (P1: *F*_1,353_ = 129.30, *P* < 0.0001; latency: *LRT* = 213.03, *P* < 0.0001). Our failure to find an association between these postmating phenotypes and competition-dependent variation in SFP transfer is surprising given the mechanistic link between these phenotypes and SFPs, and given a previous study demonstrating a sensitivity of these phenotypes to rival exposure (39; but see ref. 40).

We hypothesized that our failure to find a difference may relate to the 3-d intermating interval we allowed for. The effect of competition could be stronger with shorter intermating intervals, where fewer stored sperm have been used. However, we found that when males were singly mated to females that remated 1 d later, there was still no significant effect of level of competition on defensive sperm competition performance (*F*_2,194_ = 0.06, *P* = 0.942; *SI Appendix*, Fig. S5*A*) or female receptivity to remating (*LRT* = 0.45, *P* = 0.799; *SI Appendix*, Fig. S5*B*), and surprisingly no significant difference in the number of offspring produced in the 24 h following mating (*F*_2,836_ = 0.45, *P* = 0.636; *SI Appendix*, Fig. S5*C*), despite a significant increase in mating duration (*F*_2,861_ = 44.046, *P* < 0.0001; *SI Appendix*, Fig. S5*D*). Greater offspring production induced by the ejaculates of high-competition males over 3 d postmating (Fig. 4), but not over 1, suggests that this change is driven by longer term-acting SFPs, not short-term fecundity stimulators (e.g., ovulin). This is consistent with our finding that many sex peptide network proteins (such as antr, aqrs, and CG1652) are transferred in greater abundance by males in the high-competition treatment. Sex peptide is bound to sperm upon mating, and these sex peptide network proteins are required for the gradual release of sex peptide in the days that follow—a process that regulates long-term sperm use and stimulation of offspring production (60).

## Conclusion

Our data highlight the remarkable sensitivity of males to the social environment. Importantly, we show that the sperm allocation response to competition diverges from compositional changes in the seminal fluid. The mechanisms by which males translate social stimulation into differentially composed ejaculates are unclear. One possibility is that this ability may be facilitated by interactions between accessory gland cells and a neuropeptide circuit known to coordinate the transfer of sperm and seminal fluid (70, 74). Recent work has also indicated that some cellular processes in accessory glands are mediated by the hormone ecdysone, the concentration of which is known to be sensitive to sociosexual conditions (75, 76). Our work further shows that ejaculate compositional change is associated with costs to further matings, indicating that males are under selection to optimize tradeoffs between current and future mating opportunities. In order to better resolve the adaptive significance of this plasticity, we must now establish how male perception of competition levels is influenced by changes in the social environment, and how changes in the intensity of male–male competition modify the mating context males expect to face; for example, how does realized sperm competition intensity change as a function of the number of rivals? Collectively, our findings have broad implications for other taxa. Shedding light onto the physiological and behavioral mechanisms underpinning seminal fluid allocation, for example, may provide novel opportunities for therapeutic interventions that modify seminal fluid components known to affect fertility, interact with the female reproductive tract, or affect offspring development (21, 22). The ability of males to engineer ejaculate composition could be harnessed for a diverse range of assisted reproductive technology applications relating to livestock production, conservation strategies, and human reproductive health.

## Materials and Methods

### Fly Stocks and Husbandry.

Males were from a laboratory-adapted, outbred Dahomey wild-type stock (kept at large population size with overlapping generations) that has been repeatedly used in male reproductive plasticity studies (31, 34, 39, 40). Into this background, we backcrossed *GFP-ProtB*, courtesy of Stefan Lüpold, University of Zürich, Zürich, Switzerland, to fluorescently label sperm heads (77) for our sperm count experiment and, separately, *sparkling*^*poliert*^ (*spa*^*pol*^) for all of our competitor males and females. *spa*^*pol*^ provides a recessive phenotypic marker for paternity assignment. All flies were kept in a nonhumidified room at 25 °C on a 12:12-h L:D cycle, fed on Lewis medium, and reared at standardized larval densities by transferring ∼200 eggs to 250-mL bottles containing 50 mL food (as in ref. 78). Experimental males were collected as virgins (i.e., within 7 h of eclosion) under ice anesthesia and randomly placed either individually, in same-sex pairs, or in a same-sex group of 8 in 36-mL Lewis medium-containing plastic vials. Here, they were aged for 3 to 4 d before use in mating experiments. All females and the competitor males used in competition assays were collected as virgins, and held in groups of 10 to 12. Females were 3 to 4 d old at time of first mating while competitor males were 4 to 5 d old. All experimental flies were supplemented with ad libitum live yeast.

### Mating Experiments.

Single males from the three competition treatments were aspirated into yeasted vials containing virgin females individually isolated the evening before. For all matings, we recorded the time the male entered the vial, the time mating began, and the time it ended, from which we calculated duration of and latency to mating. For sperm counts, females were flash-frozen in liquid nitrogen 25 min after mating began and subsequently stored at −80 °C until dissection. This experiment was conducted in 2 blocks. For the 5-mating experiments, females were removed from the vial after the end of mating before the introduction of each new virgin. Only a male’s first and fifth partners were retained for analysis of postmating phenotypes. These females were transferred into fresh vials every 24 h for 3 d. On the fourth day, we aspirated mated females into vials containing two competitor males paired the evening before. After mating, males were removed and the female was transferred onto fresh food every 24 h for 4 d. Females not remating within 420 min were censored. For the accessory gland measurement analysis, males were flash-frozen in liquid nitrogen at preassigned time points after their fifth mating (0, 12, 24, 36, 60 h) and stored at −80 °C until dissection. This experiment was performed in 2 blocks. For the short intermating interval experiment the same protocol was used except males had a single mating and females were offered a remating 24 h later. This experiment was performed in 5 blocks. Offspring-containing vials in all experiments were frozen after the flies eclosed and stored at −20 °C until counting.

### Sperm Counts.

Female reproductive tracts were dissected in ice-cold PBS under a light microscope. We sealed the coverslip in place with rubber cement (Fixogum; Marabu) and imaged the sample with a Zeiss 880 confocal microscope. We used an average-intensity *Z* projection in ImageJ (79) to condense *Z* stacks into a single image to facilitate counting, which we performed manually using the multipoint feature.

### Accessory Gland Measurements.

Accessory glands were dissected in ice-cold PBS under a light microscope and photographed using a Chromyx HD camera under bright-field microscopy (Motic; BA210) at 10× magnification. We then traced the outline of each lobe and measured the internal area (summed across the 2 lobes) in ImageJ (79). Images where one gland was punctured were omitted from analysis.

### Proteomics Experiment.

Males were either introduced into a female-containing vial or into a paired vacant vial to be retained as a virgin. Twenty-five minutes after the start of mating, we aspirated the newly mated male into a cryovial before flash-freezing in liquid nitrogen. We simultaneously froze the virgin male in the partner vial. Thus, the distribution of freezing times among virgin and mated males was equivalent. Freezing males at 25 min after the start of mating represents a time point very soon after the end of mating, where mating typically lasts ∼20 min, and is consistent with previous work (4, 31, 46). Males were stored at −80 °C until dissection. This experiment was conducted 5 times to produce 5 independent biological replicates. When dissecting accessory glands, we severed the ejaculatory duct at the distal end, removing the seminal vesicles and testes. Each sample was composed of 20 pairs of glands pooled in 25 µL of PBS. Factoring in the 5 replicates, we had 30 samples in total, which we held at −80 °C. Our quantitative proteomics analysis was conducted in accordance with the gel-aided sample preparation protocol (46, 80). Details of this method, the LC-MS/MS platform, and the data processing and normalization are given in *SI Appendix*.

The mass spectrometry proteomics data were deposited in the ProteomeXchange Consortium via the PRIDE (81) partner repository with the dataset identifier PXD009451 [specifically, the “male dataset 2” subset; Sepil et al. (46)]. All other datasets are publicly available in the Oxford University Research Archive (https://ora.ox.ac.uk; DOI: 10.5287/Bodleian:zBdPnBZNB).

### Data Analysis.

All statistical analyses were performed with R statistical software (version 3.5.1). Sperm transfer lasts ∼1 min and is complete by 8 min after the start of mating (73, 82). Therefore, we excluded the small number of males for which any of their mating durations fell outside of 8 ≤ *t* ≤ 39. In all analyses, we used Grubbs’s test to detect extreme outliers (83). Across all datasets, the outliers detected were each of an extremely high and low sperm count and 2 extremely low offspring production differentials, which we Winsorized in both cases (84). In all models, we assessed fit by visual inspection of diagnostic plots (85) and the significance of factors by dropping individual terms from the full model using the “drop1” function, refitting where the interaction was nonsignificant. Replicate was always included as a fixed effect, due to there being <6 levels (86). Sperm count data were square-transformed and analyzed by linear model. Accessory gland size data were log-transformed and analyzed by linear model. In all but this case, we plot untransformed data as we believe the raw values will be of interest. Mating latency data were analyzed through Cox proportional hazard models using the *survival* and *survminer* packages (87–89). Data were censored according to whether the male/female mated. Sperm competition data were analyzed by generalized linear model (GLM) with a quasibinomial distribution to account for overdispersion (86). For paternity share analyses, we included data only from individuals that produced at least one offspring from each male at some point after the first mating to focus on females that received sperm from both males. We square-transformed offspring counts and, in the 5-mating experiment, analyzed them using a linear mixed effects model that included male identity as a random effect. Males were excluded if they failed to produce offspring in all of their matings. The *P* values from linear mixed effects models, which we also used to analyze mating duration, were calculated using Satterthwaite’s method (86). When analyzing the offspring production differential, we subtracted the fifth-mating offspring total from the first-mating total for each male. In all cases, post hoc pairwise comparisons were performed using the *lsmeans* package without *P-*value correction (90).

We conducted all proteomics analysis on log_2_-transformed abundances. To restrict analysis to proteins with high-confidence quantitation, we excluded proteins detected with fewer than 2 unique peptides (37, 91). Proteins were described as SFPs if known to be transferred to females, based on a reference list provided by Mariana Wolfner, Cornell University, Ithaca, NY, and Geoff Findlay, College of the Holy Cross, Worcester, MA, and updated to include high-confidence SFPs from Sepil et al. (46). We also included intrepid (intr), despite it not having been conclusively shown to be transferred to females, as we find it at significantly lower abundance in mated glands and given it has known roles in the sex peptide network (59). Variables in our PCAs were scaled to have unit variance and shifted to be zero-centered. We extracted scores for the first three PCs from the PCA data frame to which we then fitted a linear model. We took an average across 5 replicates for each protein in the 6 treatment combinations (competition x mating) for our clustering analysis, which used a Pearson correlation distance metric, and plotted the output as a heatmap using the *pheatmap* package (92). Major clusters were identified by visual inspection. For visualization of relative abundance patterns, we divided each protein’s normalized abundance value by the mean across all 30 samples for that protein (“mean centering”). This allows for comparison between different SFPs across the dynamic range of SFP abundances. Clusters were analyzed by linear mixed effects models, with protein identity as a random effect, and mating status and competition level as fixed effects. For our differential SFP abundance analysis, we iterated a linear model over all detected proteins across the 30 samples, including competition level, replicate, and mating status as factors. We used a tail-based false discovery rate correction from the *fdrtool* package (93). Pairwise log_2_ fold changes use the mean across replicates for each individual SFP within a treatment combination. Fold changes are calculated according to χ_i,j_ = χ_j_ − χ_i_, where χ is virgin or mated and *i* and *j* are the group sizes being compared.

## Supplementary Material

Supplementary File

## References

[1] Dewsbury D. A., Ejaculate cost and male choice. Am. Nat. 119, 601–610 (1982).

[2] Dewsbury D. A., The Darwin-Bateman paradigm in historical context. Integr. Comp. Biol. 45, 831–837 (2005). 21676834 10.1093/icb/45.5.831

[3] Reinhardt K., Naylor R., Siva-Jothy M. T., Male mating rate is constrained by seminal fluid availability in bedbugs, *Cimex lectularius*. PLoS One 6, e22082 (2011). 21779378 10.1371/journal.pone.0022082 PMC3136940

[4] Sirot L. K., Buehner N. A., Fiumera A. C., Wolfner M. F., Seminal fluid protein depletion and replenishment in the fruit fly, *Drosophila melanogaster*: An ELISA-based method for tracking individual ejaculates. Behav. Ecol. Sociobiol. 63, 1505–1513 (2009). 24733957 10.1007/s00265-009-0806-6 PMC3984576

[5] Wedell N., Gage M. J. G., Parker G. A., Sperm competition, male prudence and sperm-limited females. Trends Ecol. Evol. 17, 313–320 (2002).

[6] Preston B. T., Stevenson I. R., Pemberton J. M., Wilson K., Dominant rams lose out by sperm depletion. Nature 409, 681–682 (2001). 11217847 10.1038/35055617

[7] Boivin G., Jacob S., Damiens D., Spermatogeny as a life-history index in parasitoid wasps. Oecologia 143, 198–202 (2005). 15657761 10.1007/s00442-004-1800-3

[8] Vahed K., Parker D. J., Gilbert J. D. J., Larger testes are associated with a higher level of polyandry, but a smaller ejaculate volume, across bushcricket species (Tettigoniidae). Biol. Lett. 7, 261–264 (2011). 21068028 10.1098/rsbl.2010.0840 PMC3061181

[9] Friesen C. R., Powers D. R., Copenhaver P. E., Mason R. T., Size dependence in non-sperm ejaculate production is reflected in daily energy expenditure and resting metabolic rate. J. Exp. Biol. 218, 1410–1418 (2015). 25954044 10.1242/jeb.120402

[10] Parker G. A., Pizzari T., Sperm competition and ejaculate economics. Biol. Rev. Camb. Philos. Soc. 85, 897–934 (2010). 20560928 10.1111/j.1469-185X.2010.00140.x

[11] Parker G. A., Ball M. A., Stockley P., Gage M. J. G., Sperm competition games: A prospective analysis of risk assessment. Proc. Biol. Sci. 264, 1793–1802 (1997). 9447737 10.1098/rspb.1997.0249 PMC1688741

[12] Parker G. A., Sperm competition games: Sneaks and extra-pair copulations. Proc. Biol. Sci. 242, 127–133 (1990).

[13] Kelly C. D., Jennions M. D., Sexual selection and sperm quantity: Meta-analyses of strategic ejaculation. Biol. Rev. Camb. Philos. Soc. 86, 863–884 (2011). 21414127 10.1111/j.1469-185X.2011.00175.x

[14] Parker G. A., Ball M. A., Stockley P., Gage M. J. G., Sperm competition games: Individual assessment of sperm competition intensity by group spawners. Proc. Biol. Sci. 263, 1291–1297 (1996).

[15] Tazzyman S. J., Pizzari T., Seymour R. M., Pomiankowski A., The evolution of continuous variation in ejaculate expenditure strategy. Am. Nat. 174, E71–E82 (2009). 19627229 10.1086/603612

[16] Williams P. D., Day T., Cameron E., The evolution of sperm-allocation strategies and the degree of sperm competition. Evolution 59, 492–499 (2005). 15856692

[17] Fromhage L., McNamara J. M., Houston A. I., Sperm allocation strategies and female resistance: A unifying perspective. Am. Nat. 172, 25–33 (2008). 18500937 10.1086/587806

[18] Poiani A., Complexity of seminal fluid: A review. Behav. Ecol. Sociobiol. 60, 289–310 (2006).

[19] Hopkins B. R., Sepil I., Wigby S., Seminal fluid. Curr. Biol. 27, R404–R405 (2017). 28586660 10.1016/j.cub.2017.03.063

[20] Avila F. W., Sirot L. K., LaFlamme B. A., Rubinstein C. D., Wolfner M. F., Insect seminal fluid proteins: Identification and function. Annu. Rev. Entomol. 56, 21–40 (2011). 20868282 10.1146/annurev-ento-120709-144823 PMC3925971

[21] Robertson S. A., Sharkey D. J., Seminal fluid and fertility in women. Fertil. Steril. 106, 511–519 (2016). 27485480 10.1016/j.fertnstert.2016.07.1101

[22] McGraw L. A., Suarez S. S., Wolfner M. F., On a matter of seminal importance. BioEssays 37, 142–147 (2015). 25379987 10.1002/bies.201400117 PMC4502980

[23] Hodgson D. J., Hosken D. J., Sperm competition promotes the exploitation of rival ejaculates. J. Theor. Biol. 243, 230–234 (2006). 16901507 10.1016/j.jtbi.2006.06.024

[24] Cameron E., Day T., Rowe L., Sperm competition and the evolution of ejaculate composition. Am. Nat. 169, E158–E172 (2007). 17479456 10.1086/516718

[25] Fedorka K. M., Winterhalter W. E., Ware B., Perceived sperm competition intensity influences seminal fluid protein production prior to courtship and mating. Evolution 65, 584–590 (2011). 21271997 10.1111/j.1558-5646.2010.01141.x

[26] Sloan N. S., Lovegrove M., Simmons L. W., Social manipulation of sperm competition intensity reduces seminal fluid gene expression. Biol. Lett. 14, 20170659 (2018). 29367215 10.1098/rsbl.2017.0659 PMC5803594

[27] Simmons L. W., Lovegrove M., Socially cued seminal fluid gene expression mediates responses in ejaculate quality to sperm competition risk. Proc. Biol. Sci. 284, 20171486 (2017). 28855372 10.1098/rspb.2017.1486 PMC5577498

[28] Nakadera Y., Giannakara A., Ramm S. A., Plastic expression of seminal fluid protein genes in a simultaneously hermaphroditic snail. Behav. Ecol. 30, 904–913 (2019).

[29] Patlar B., Weber M., Ramm S. A., Genetic and environmental variation in transcriptional expression of seminal fluid proteins. Heredity 122, 595–611 (2019). 30356222 10.1038/s41437-018-0160-4 PMC6461930

[30] Pizzari T., Cornwallis C. K., Løvlie H., Jakobsson S., Birkhead T. R., Sophisticated sperm allocation in male fowl. Nature 426, 70–74 (2003). 14603319 10.1038/nature02004

[31] Sirot L. K., Wolfner M. F., Wigby S., Protein-specific manipulation of ejaculate composition in response to female mating status in *Drosophila melanogaster*. Proc. Natl. Acad. Sci. U.S.A. 108, 9922–9926 (2011). 21628597 10.1073/pnas.1100905108 PMC3116428

[32] Khan Z., Primate transcript and protein expression levels evolve under compensatory selection pressures. Science 342, 1100–1104 (2013). 24136357 10.1126/science.1242379 PMC3994702

[33] Liu Y., Beyer A., Aebersold R., On the dependency of cellular protein levels on mRNA abundance. Cell 165, 535–550 (2016). 27104977 10.1016/j.cell.2016.03.014

[34] Wigby S., Seminal fluid protein allocation and male reproductive success. Curr. Biol. 19, 751–757 (2009). 19361995 10.1016/j.cub.2009.03.036 PMC2737339

[35] Hopkins B. R., Avila F. W., Wolfner M. F., “Insect male reproductive glands and their products” in Encyclopedia of Reproduction, Skinner M. K., Ed. (Elsevier, 2018), pp. 137–144.

[36] Rolland A. D., Identification of genital tract markers in the human seminal plasma using an integrative genomics approach. Hum. Reprod. 28, 199–209 (2013). 23024119 10.1093/humrep/des360

[37] Borziak K., Álvarez-Fernández A., Karr T. L., Pizzari T., Dorus S., The seminal fluid proteome of the polyandrous red junglefowl offers insights into the molecular basis of fertility, reproductive ageing and domestication. Sci. Rep. 6, 35864 (2016). 27804984 10.1038/srep35864 PMC5090203

[38] Ramm S. A., Sperm competition risk drives plasticity in seminal fluid composition. BMC Biol. 13, 87 (2015). 26507392 10.1186/s12915-015-0197-2 PMC4624372

[39] Bretman A., Fricke C., Chapman T., Plastic responses of male *Drosophila melanogaster* to the level of sperm competition increase male reproductive fitness. Proc. Biol. Sci. 276, 1705–1711 (2009). 19324834 10.1098/rspb.2008.1878 PMC2660996

[40] Bretman A., Westmancoat J. D., Gage M. J. G., Chapman T., Costs and benefits of lifetime exposure to mating rivals in male *Drosophila melanogaster*. Evolution 67, 2413–2422 (2013). 23888861 10.1111/evo.12125

[41] Perry J. C., Sirot L., Wigby S., The seminal symphony: How to compose an ejaculate. Trends Ecol. Evol. 28, 414–422 (2013). 23582755 10.1016/j.tree.2013.03.005 PMC4974483

[42] Dhole S., Servedio M. R., Sperm competition and the evolution of seminal fluid composition. Evolution 68, 3008–3019 (2014). 24975874 10.1111/evo.12477

[43] Bretman A., Fricke C., Hetherington P., Stone R., Chapman T., Exposure to rivals and plastic responses to sperm competition in *Drosophila melanogaster*. Behav. Ecol. 21, 317–321 (2010).

[44] Mohorianu I., Genomic responses to the socio-sexual environment in male *Drosophila melanogaster* exposed to conspecific rivals. RNA 23, 1048–1059 (2017). 28428330 10.1261/rna.059246.116 PMC5473139

[45] Bretman A., Gage M. J. G., Chapman T., Quick-change artists: Male plastic behavioural responses to rivals. Trends Ecol. Evol. 26, 467–473 (2011). 21680050 10.1016/j.tree.2011.05.002

[46] Sepil I., Quantitative proteomics identification of seminal fluid proteins in male *Drosophila melanogaster*. Mol. Cell. Proteomics 18 (suppl. 1), S46–S58 (2019). 30287546 10.1074/mcp.RA118.000831 PMC6427238

[47] Findlay G. D., Yi X., Maccoss M. J., Swanson W. J., Proteomics reveals novel *Drosophila* seminal fluid proteins transferred at mating. PLoS Biol. 6, e178 (2008). 18666829 10.1371/journal.pbio.0060178 PMC2486302

[48] Garbaczewska M., Billeter J. C., Levine J. D., *Drosophila melanogaster* males increase the number of sperm in their ejaculate when perceiving rival males. J. Insect Physiol. 59, 306–310 (2013). 23178803 10.1016/j.jinsphys.2012.08.016

[49] Moatt J. P., Dytham C., Thom M. D. F., Sperm production responds to perceived sperm competition risk in male *Drosophila melanogaster*. Physiol. Behav. 131, 111–114 (2014). 24769021 10.1016/j.physbeh.2014.04.027

[50] Huang W., Sherman B. T., Lempicki R. A., Bioinformatics enrichment tools: Paths toward the comprehensive functional analysis of large gene lists. Nucleic Acids Res. 37, 1–13 (2009). 19033363 10.1093/nar/gkn923 PMC2615629

[51] Huang W., Sherman B. T., Lempicki R. A., Systematic and integrative analysis of large gene lists using DAVID bioinformatics resources. Nat. Protoc. 4, 44–57 (2009). 19131956 10.1038/nprot.2008.211

[52] Mueller J. L., Ripoll D. R., Aquadro C. F., Wolfner M. F., Comparative structural modeling and inference of conserved protein classes in *Drosophila* seminal fluid. Proc. Natl. Acad. Sci. U.S.A. 101, 13542–13547 (2004). 15345744 10.1073/pnas.0405579101 PMC518759

[53] Mohorianu I., Fowler E. K., Dalmay T., Chapman T., Control of seminal fluid protein expression via regulatory hubs in *Drosophila melanogaster*. Proc. Biol. Sci. 285, 20181681 (2018). 30257913 10.1098/rspb.2018.1681 PMC6170815

[54] Wong A., A role for Acp29AB, a predicted seminal fluid lectin, in female sperm storage in *Drosophila melanogaster*. Genetics 180, 921–931 (2008). 18757944 10.1534/genetics.108.092106 PMC2567391

[55] Fiumera A. C., Dumont B. L., Clark A. G., Sperm competitive ability in *Drosophila melanogaster* associated with variation in male reproductive proteins. Genetics 169, 243–257 (2005). 15466425 10.1534/genetics.104.032870 PMC1448872

[56] Clark A. G., Aguadé M., Prout T., Harshman L. G., Langley C. H., Variation in sperm displacement and its association with accessory gland protein loci in *Drosophila melanogaster*. Genetics 139, 189–201 (1995). 7705622 10.1093/genetics/139.1.189 PMC1206317

[57] Ram K. R., Wolfner M. F., Sustained post-mating response in *Drosophila melanogaster* requires multiple seminal fluid proteins. PLoS Genet. 3, e238 (2007). 18085830 10.1371/journal.pgen.0030238 PMC2134937

[58] Ram K. R., Wolfner M. F., A network of interactions among seminal proteins underlies the long-term postmating response in *Drosophila*. Proc. Natl. Acad. Sci. U.S.A. 106, 15384–15389 (2009). 19706411 10.1073/pnas.0902923106 PMC2741260

[59] Findlay G. D., Evolutionary rate covariation identifies new members of a protein network required for *Drosophila melanogaster* female post-mating responses. PLoS Genet. 10, e1004108 (2014). 24453993 10.1371/journal.pgen.1004108 PMC3894160

[60] Avila F. W., Ravi Ram K., Bloch Qazi M. C., Wolfner M. F., Sex peptide is required for the efficient release of stored sperm in mated *Drosophila* females. Genetics 186, 595–600 (2010). 20679516 10.1534/genetics.110.119735 PMC2954482

[61] Gligorov D., Sitnik J. L., Maeda R. K., Wolfner M. F., Karch F., A novel function for the Hox gene Abd-B in the male accessory gland regulates the long-term female post-mating response in *Drosophila*. PLoS Genet. 9, e1003395 (2013). 23555301 10.1371/journal.pgen.1003395 PMC3610936

[62] Singh A., Long-term interaction between *Drosophila* sperm and sex peptide is mediated by other seminal proteins that bind only transiently to sperm. Insect Biochem. Mol. Biol. 102, 43–51 (2018). 30217614 10.1016/j.ibmb.2018.09.004 PMC6249070

[63] Clutton-Brock T. H., Reproductive effort and terminal investment in iteroparous animals. Am. Nat. 123, 212–229 (1984).

[64] Liu W., Social regulation of aggression by pheromonal activation of Or65a olfactory neurons in *Drosophila*. Nat. Neurosci. 14, 896–902 (2011). 21685916 10.1038/nn.2836

[65] Yuan Q., Song Y., Yang C. H., Jan L. Y., Jan Y. N., Female contact modulates male aggression via a sexually dimorphic GABAergic circuit in *Drosophila*. Nat. Neurosci. 17, 81–88 (2014). 24241395 10.1038/nn.3581 PMC3995170

[66] Lim R. S., Eyjólfsdóttir E., Shin E., Perona P., Anderson D. J., How food controls aggression in *Drosophila*. PLoS One 9, e105626 (2014). 25162609 10.1371/journal.pone.0105626 PMC4146546

[67] Sepil I., Ejaculate deterioration with male age, and its amelioration in *Drosophila*. bioRxiv:10.1101/624734 (28 June 2019).

[68] Leech T., Sait S. M., Bretman A., Sex-specific effects of social isolation on ageing in *Drosophila melanogaster*. J. Insect Physiol. 102, 12–17 (2017). 28830760 10.1016/j.jinsphys.2017.08.008

[69] Linklater J. R., Wertheim B., Wigby S., Chapman T., Ejaculate depletion patterns evolve in response to experimental manipulation of sex ratio in *Drosophila melanogaster*. Evolution 61, 2027–2034 (2007). 17683443 10.1111/j.1558-5646.2007.00157.x

[70] Tayler T. D., Pacheco D. A., Hergarden A. C., Murthy M., Anderson D. J., A neuropeptide circuit that coordinates sperm transfer and copulation duration in *Drosophila*. Proc. Natl. Acad. Sci. U.S.A. 109, 20697–20702 (2012). 23197833 10.1073/pnas.1218246109 PMC3528491

[71] Crickmore M. A., Vosshall L. B., Opposing dopaminergic and GABAergic neurons control the duration and persistence of copulation in *Drosophila*. Cell 155, 881–893 (2013). 24209625 10.1016/j.cell.2013.09.055 PMC4048588

[72] Lüpold S., Manier M. K., Ala-Honkola O., Belote J. M., Pitnick S., Male *Drosophila melanogaster* adjust ejaculate size based on female mating status, fecundity, and age. Behav. Ecol. 22, 185–191 (2011).

[73] Gilchrist A. S., Partridge L., Why it is difficult to model sperm displacement in *Drosophila melanogaster*: The relation between sperm transfer and copulation duration. Evolution 54, 534–542 (2000). 10937230 10.1111/j.0014-3820.2000.tb00056.x

[74] Redhai S., Regulation of dense-core granule replenishment by autocrine BMP signalling in *Drosophila* secondary cells. PLoS Genet. 12, e1006366 (2016). 27727275 10.1371/journal.pgen.1006366 PMC5065122

[75] Wilson C., Mating induces switch from hormone-dependent to –independent steroid receptor-mediated growth in *Drosophila* prostate-like cells. bioRxiv:10.1101/533976 (29 January 2019).

[76] Sharma V., Functional male accessory glands and fertility in *Drosophila* require novel ecdysone receptor. PLoS Genet. 13, e1006788 (2017). 28493870 10.1371/journal.pgen.1006788 PMC5444863

[77] Manier M. K., Resolving mechanisms of competitive fertilization success in *Drosophila melanogaster*. Science 328, 354–357 (2010). 20299550 10.1126/science.1187096

[78] Clancy D. J., Kennington W. J., A simple method to achieve consistent larval density in bottle culture. Drosoph. Inf. Serv. 84, 168–169 (2001).

[79] Schindelin J., Fiji: An open-source platform for biological-image analysis. Nat. Methods 9, 676–682 (2012). 22743772 10.1038/nmeth.2019 PMC3855844

[80] Fischer R., Kessler B. M., Gel-aided sample preparation (GASP)—A simplified method for gel-assisted proteomic sample generation from protein extracts and intact cells. Proteomics 15, 1224–1229 (2015). 25515006 10.1002/pmic.201400436 PMC4409837

[81] Vizcaíno J. A., 2016 update of the PRIDE database and its related tools. Nucleic Acids Res. 44, D447–D456 (2016). 26527722 10.1093/nar/gkv1145 PMC4702828

[82] Garcia-Bellido A., Das secret der Paragonien als Stimulus der Fekundität bei Weibachen von *Drosophila melanogaster* [in German]. Z. Naturforsch. B 19, 491–495 (1964). 14251210

[83] Grubbs F. E., Sample criteria for testing outlying observations. Ann. Math. Stat. 21, 27–58 (1950).

[84] Tukey J. W., The future of data analysis. Ann. Math. Stat. 33, 1–67 (1962).

[85] Zuur A. F., Ieno E. N., Elphick C. S., A protocol for data exploration to avoid common statistical problems. Methods Ecol. Evol. 1, 3–14 (2010).

[86] Bolker B. M., Generalized linear mixed models: A practical guide for ecology and evolution. Trends Ecol. Evol. 24, 127–135 (2009). 19185386 10.1016/j.tree.2008.10.008

[87] Therneau T. M., Grambsch P. M., Modeling Survival Data: Extending the Cox Model (Springer, New York, 2000).

[88] Therneau T., A Package for Survival Analysis in S (Version 2.38, R Package, 2015).

[89] Kassambara A., Kosinski M., survminer: Drawing Survival Curves Using “ggplot2” (Version 0.4.3, R Package, 2018).

[90] Lenth R. V., Least-squares means: The R package lsmeans. J. Stat. Softw. 69, 1–33 (2016).

[91] Carr S.; Working Group on Publication Guidelines for Peptide and Protein Identification Data, The need for guidelines in publication of peptide and protein identification data: Working Group on Publication Guidelines for Peptide and Protein Identification Data. Mol. Cell. Proteomics 3, 531–533 (2004). 15075378 10.1074/mcp.T400006-MCP200

[92] Kolde R., pheatmap: Pretty Heatmaps (Version 1.0.10, R Package, 2018).

[93] Strimmer K., fdrtool: A versatile R package for estimating local and tail area-based false discovery rates. Bioinformatics 24, 1461–1462 (2008). 18441000 10.1093/bioinformatics/btn209

[94] Laflamme B. A., Avila F. W., Michalski K., Wolfner M. F., A *Drosophila* protease cascade member, seminal metalloprotease-1, is activated stepwise by male factors and requires female factors for full activity. Genetics 196, 1117–1129 (2014). 24514904 10.1534/genetics.113.160101 PMC3982676

