# Supplementary material for: Divergent allocation of sperm and the seminal proteome along a competition gradient in Drosophila melanogaster
Source: Proc Natl Acad Sci U S A. 2019 Aug 20;116(36):17925–33. doi: 10.1073/pnas.1906149116 (PMC6731677; doi:10.1073/pnas.1906149116)
Supplement: Supplementary File [file pnas.1906149116.sapp.pdf]

PNAS
www.pnas.org

Supplementary Information for

Divergent allocation of sperm and the seminal proteome along a competition gradient in *Drosophila melanogaster*

Ben R. Hopkins, Irem Sepil, Marie-Laëtitia Thézénas, James F. Craig, Thomas Miller, Philip D. Charles, Roman Fischer, Benedikt M. Kessler, Amanda Bretman, Tommaso Pizzari, and Stuart Wigby

Corresponding author:
Ben R. Hopkins

Email: brhopkins92@gmail.com

This PDF file includes:

Supplementary information text
References for SI
Figs. S1 to S5
Tables S1 to S5

www.pnas.org/cgi/doi/10.1073/pnas.1906149116

Supplementary Information Text

Materials and Methods

Proteomics sample preparation

To prepare the sample for proteomic analysis, we used a clean pestle to macerate the pooled glands in each sample for 1-minute, washing the residue from the pestle back into the sample with 25µl of Pierce RIPA Buffer. Our samples were digested in accordance with the gel-aided sample preparation (GASP) protocol outlined in detail elsewhere (1,2). Briefly, 50mM of the reducing agent DTT was added to the sample, incubating for 10 to 20 minutes. To the resulting lysate, we added an equal volume of 40% acrylamide/Bis solution (37.5:1, National Diagnostics), which was subsequently left for 30 minutes at room temperature to promote the alkylation of cysteine to propionamide. To induce acrylamide polymerization and form a gel plug, we next added 5µl of 10% APS and an equivalent quantity of TEMED. The gel plug was then shredded via centrifugation through a membrane-less Spin-X filter insert (CLS9301, Sigma/Corning). The resulting gel fragments were then fixed in 5% acetic acid/40% ethanol prior to two consecutive episodes of buffer exchange with 50mM ammonium bicarbonate, 1.5M Urea, and 0.5M Thiourea. These were subsequently removed with ACN. Digestion of the immobilised proteins was carried out overnight through the action of trypsin (Promega). The resulting peptides were extracted via two repeated ACN replacements, dried, desalted in Sola SPE columns (Thermo), and then suspended in 0.1% FA, 2% ACN before LC-MS/MS (liquid chromatography- mass spectrometry/mass spectrometry) analysis.

LC-MS/MS

Peptides were analysed using a LC-MS/MS platform composed of a Dionex Ultimate 3000 and a Q-Exactive mass spectrometer (Thermo). Peptides were loaded on a trap column (PepMAP C18, 300µm x 5m, 5µm particle, Thermo) in a solution of 0.1% TFA in 2% ACN and then separated on an easy spray column (PepMAP C18, 75µm x 500m, 2µm particle, Thermo) with a gradient 2% ACN to 35% ACN in 0.1% FA in 5% DMSO. MS spectra were collected at a resolution of 70,000 in profile mode on the Q-Exactive (ion target = $3x10^6$). The 15 most intense features were selected for subsequent MS/MS analysis at a resolution of 17,500. The following parameters were set: dynamic exclusion = 27 seconds; AGC target = $1x10^5$; isolation width = 1.6 m/z; and maximum acquisition time = 128ms.

MS data processing

The MS data processing pipeline we used has previously been described by Sepil *et al.* (2). We imported the RAW data into Progenesis QIP (version 3.0.6039.34628), exporting spectra as MGF files using the 200 most intense peaks without deconvolution for searching. For peptide identification, we used the *Drosophila melanogaster* UniProt reference proteome as a search target, with database retrieval conducted on 15/02/2017 (23302 sequences) in Mascot 2.5.1. Our search parameters incorporated the following: Oxidation

(M), Propionamide (K), and Deamidation (N,Q) as variable modifications; Propionamide (C) as a fixed modification; two missed cleavage sites; 0.05 Da fragment mass accuracy; 10ppm precursor accuracy. Prior to importing the search results into Progenesis for quantification via the Top3 method, we applied a peptide-level 1% FDR alongside an additional Mascot ion score cut-off of 20. The resulting protein abundance data was subsequently normalised using the internal Progenesis algorithm to all proteins.

Supplementary References

1. Fischer R, Kessler BM (2015) Gel-aided sample preparation (GASP)-A simplified method for gel-assisted proteomic sample generation from protein extracts and intact cells. *Proteomics* 15(7):1224–1229.
2. Sepil I, et al. (2019) Quantitative proteomics identification of seminal fluid proteins in male Drosophila melanogaster. *Mol Cell Proteomics* 18(Supplement 1):S46–S58.

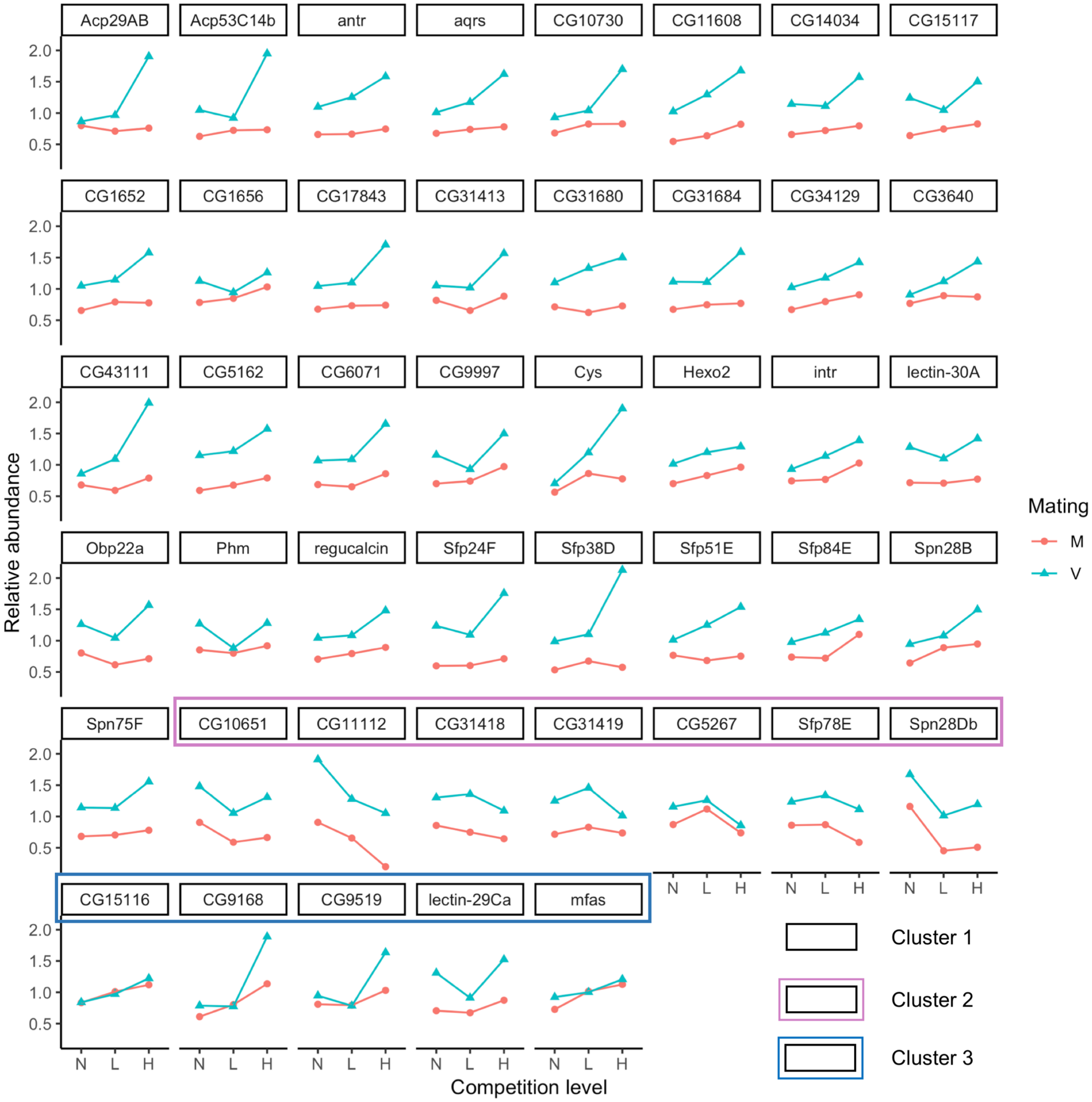

Fig. S1. Abundance profiles of SFPs identified as differentially-abundant in relation to competition intensity (FDR $p<0.05$). Each point represents an average across the 5 replicates in relation to competition level (males held alone, none, N; males held in same-sex pairs, low, L; and males held in a same-sex group of 8, high, H) and separately for mated (red) and virgin (blue) glands. The abundance values are relativized by means-centring and averaging across replicates. Coloured boxes denote membership of clusters identified in Fig. 2*A*: no box, Cluster 1; purple, Cluster 2; blue, Cluster 3.

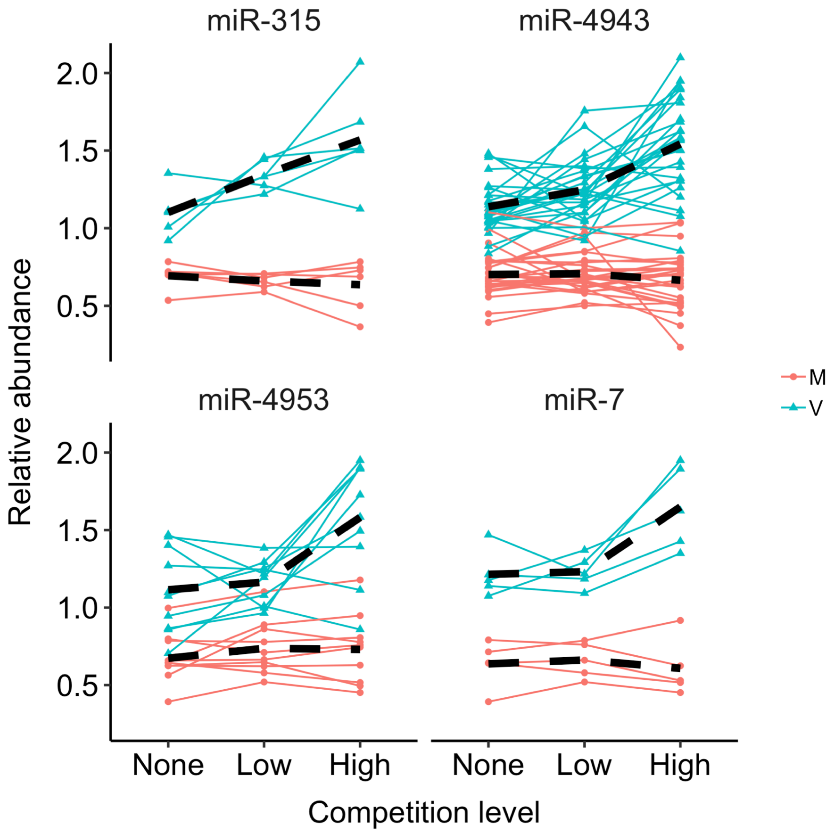

Figure S2. No evidence for different abundance patterns between groups of SFPs thought to be regulated by particular miRNAs. Average abundance patterns for each of the detected SFPs for which a putative miRNA regulator has been proposed (see main text) plotted in relation to competition level, the miRNA suggested to regulate it, and separately for mated (red) and virgin (blue) glands. The abundance values are relativized by means-centring and averaging across replicates. An average response is given in dashed black lines. Where a SFP has multiple putative miRNA regulators it is plotted separately for each. We found no significant association between miRNA and SFP abundance. See Table S4 for associated statistics.

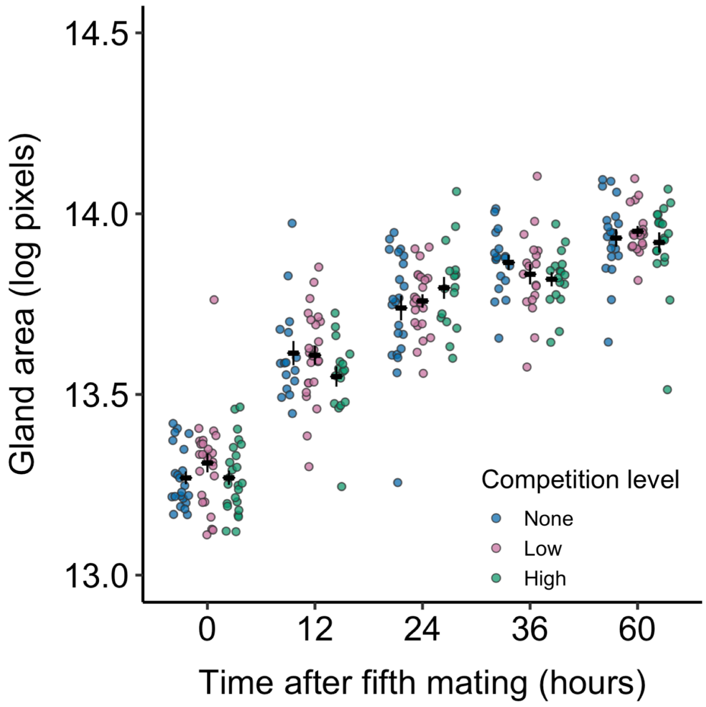

Figure S3. Accessory gland replenishment rate is unaffected by exposure to competition. Males were held alone (no competition), in a same-sex pair (low competition), or in a same-sex group of 8 (high competition) before mating to 5 virgin females. Accessory glands were dissected at one of 5 time points after the fifth mating. Gland area is summed across both lobes and is measured in log pixels. Data pooled from two replicates. *n*=18 to 26 per competition treatment per timepoint.

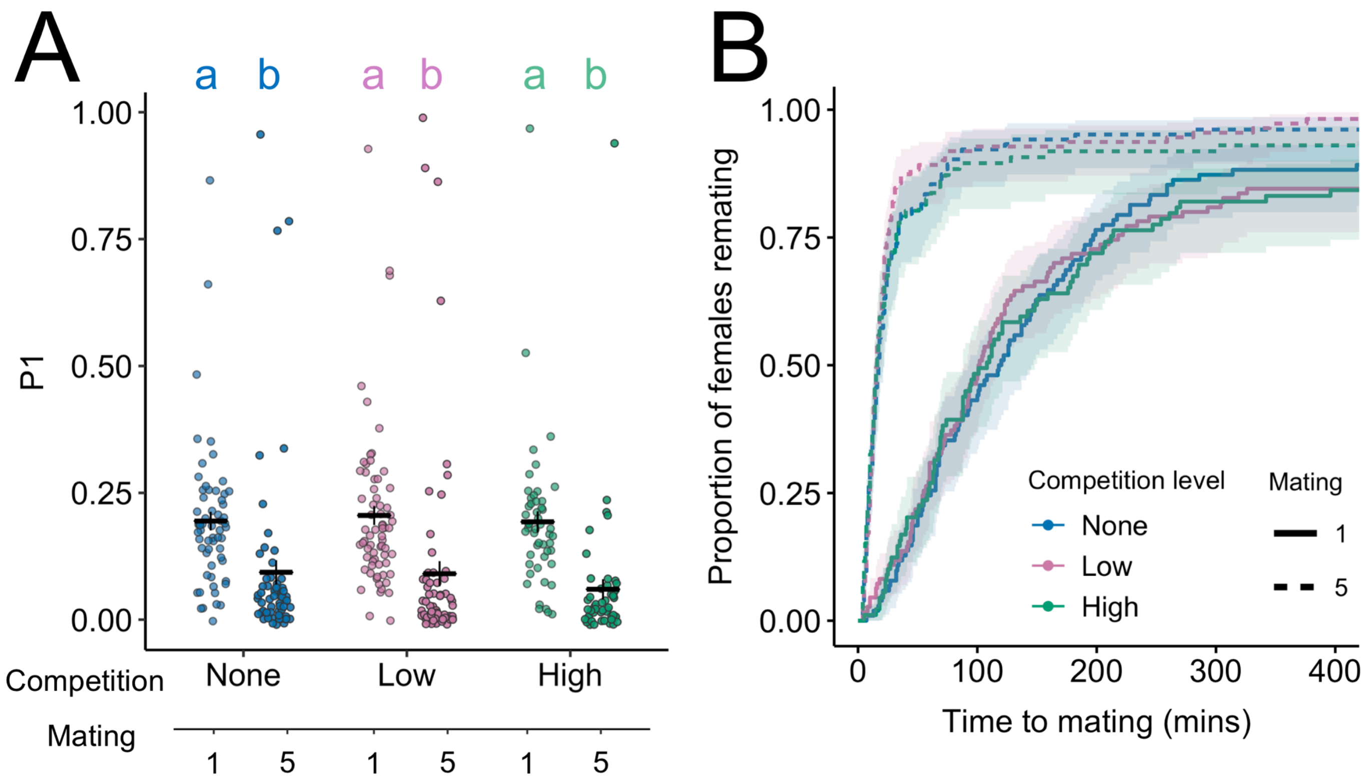

Figure S4. Significant effects of multiple mating, but not competition, on male paternity share (P1) and partner receptivity to remating. Males were held alone (no competition), in a same-sex pair (low competition), or in a same-sex group of 8 (high competition) before mating to 5 virgin females. Only females from the first and fifth matings were retained for analysis. Females remate to a standardised competitor 3 days later. Data pooled from two replicates. (*A*) First male paternity share after female remating. Mean ± SE given. $n_{no} = 60$, $n_{low} = 68$, $n_{high} = 50$. Letters give significant pairwise comparisons at $p<0.05$. (*B*) Female latency to remating. Confidence interval given at 95%. $n_{no} = 103$, $n_{low} = 112$, $n_{high} = 89$.

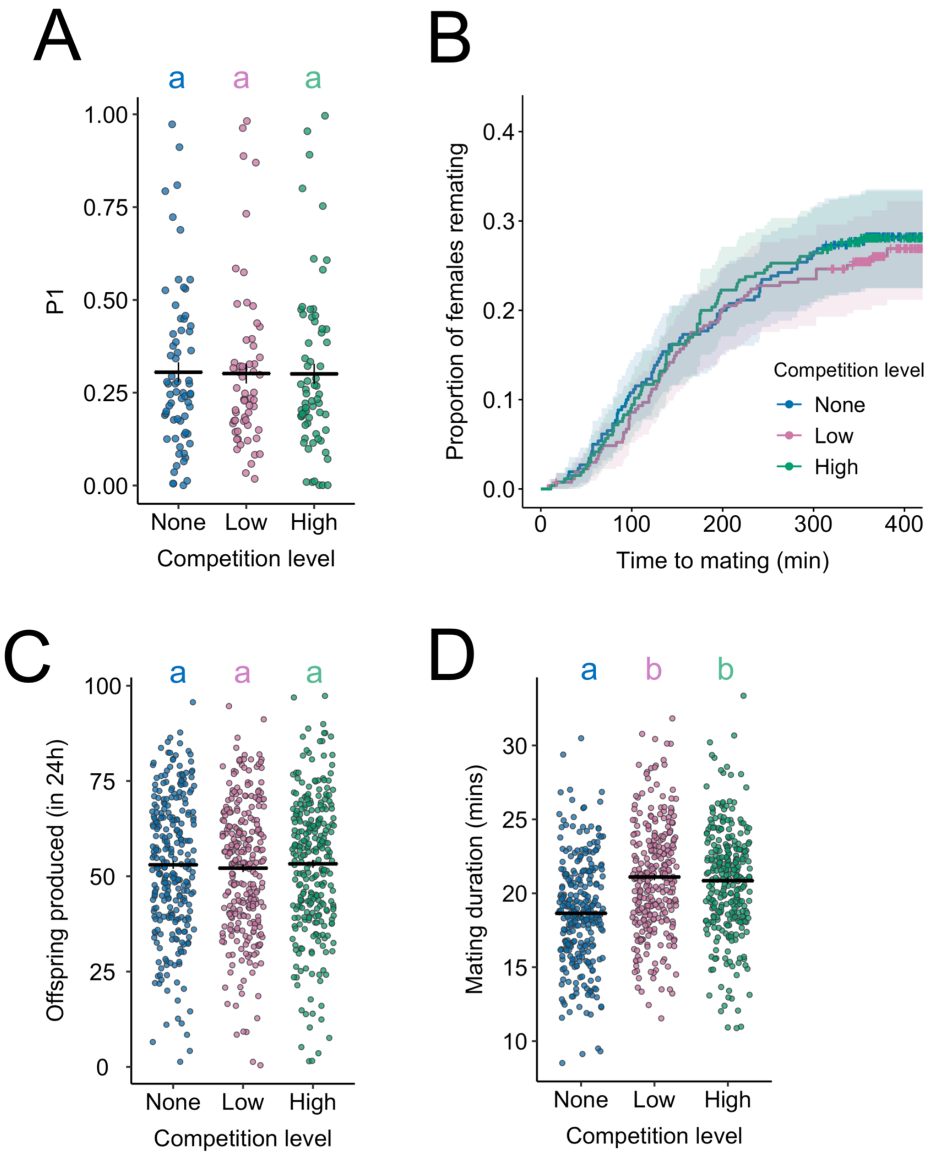

Figure S5. No effect of competition on female post-mating responses with a shortened inter-mating interval. Males were held alone (no competition), in a same-sex pair (low competition), or in a same-sex group of 8 (high competition) before mating with 1 virgin female. Females remate to a standardised competitor 24 hours later. Data pooled from 5 independent replicates. (*A*) First male paternity share after female remating. (*B*) Time to remating (in minutes) and the proportion of females that remated. Non-maters are censored. Confidence interval given at 95%. (*C*) Offspring produced in 24 hours following first mating. (*D*) Male mating duration (in minutes). In panels *A,C*, and *D,* lines give treatment mean ± 1 SE. Letters give significant pairwise comparisons at p<0.05 level. Sample sizes: *A,* n_{no} = 67, n_{low} = 63, n_{high} = 67; *B,* n_{no} = 260, n_{low} = 269, n_{high} = 266; *C,* n_{no} = 279, n_{low} = 281, n_{high} = 283; *D,* n_{no} = 288, n_{low} = 289, n_{high} = 291. Letters give significant pairwise comparisons at p<0.05.

Table S1. Cluster-specific responses of SFPs to mating and competition level. Summary statistics from least-square means post-hoc comparisons between competition levels (none, 1 male; low, 2 males; high, 8 males), within mating treatments (M=mated, V=virgin). Conducted separately for Clusters 1 and 2, which are described in Fig. 2*A,B*. Significant values at the $p<0.05$ level are given in red. Estimates are on a $\log_2$ scale.

Cluster	*Mating*	*Competition*	*Estimate*	*SE*	*DF*	*T ratio*	*p value*
1	M	None - Low	-0.065	0.038	350	-1.689	0.0921
1	M	None - High	-0.148	0.038	350	-3.878	0.0001
1	M	Low - High	-0.084	0.038	350	-2.189	0.0292
1	V	None - Low	-0.058	0.038	350	-1.507	0.1327
1	V	None - High	-0.544	0.038	350	-14.224	<0.0001
1	V	Low - High	-0.486	0.038	350	-12.717	<0.0001
2	M	None - Low	0.046	0.083	170	0.548	0.5845
2	M	None - High	0.413	0.083	170	4.952	<0.0001
2	M	Low - High	0.367	0.083	170	4.404	<0.0001
2	V	None - Low	0.029	0.083	170	0.346	0.7300
2	V	None - High	0.017	0.083	170	0.198	0.8432
2	V	Low - High	-0.012	0.083	170	-0.148	0.8828

Table S2. Summary statistics from a PCA conducted on detected SFPs. (A) Variance, eigenvalue, and loadings associated with the first four principal components (PCs). (B) Output from linear models fitted to the extracted scores from each of the first three PCs, using the measured variables of competition (none, 1 male; low, 2 males; high, 8 males), mating status (mated or virgin), and replicate (5 in total). Significant associations at the $p<0.05$ levels are given in red.

A

	PC1	*PC2*	*PC3*	*PC4*
Variance explained (%)	52.82	9.00	8.31	5.94
Eigenvalue	62.85	10.71	9.88	7.07
No. positive loadings	115	59	66	62
No. negative loadings	4	60	53	57

B

PC1 Effect	*Df*	*Sum of sq*	*RSS*	*F*	*P*
*Competition*Mating*	2	59.884	59.884	4.9385	0.0181
Competition	2	84.83	265.98	5.1514	0.0146
Mating	1	1500.98	1682.13	182.2929	<0.0001
Replicate	4	55.72	236.87	1.6919	0.1877

PC2 Effect	*Df*	*Sum of sq*	*RSS*	*F*	*P*
*Competition*Mating*	2	4.9199	115.15	0.4273	0.6581
Competition	2	139.590	259.66	12.7883	0.0002
Mating	1	4.447	124.52	0.8148	0.3765
Replicate	4	46.603	166.67	2.1347	0.1105

PC3 Effect	*Df*	*Sum of sq*	*RSS*	*F*	*P*
*Competition*Mating*	2	29.624	174.85	1.6943	0.2091
Competition	2	6.672	211.15	0.3590	0.7024
Mating	1	2.358	206.83	0.2537	0.6195
Replicate	4	73.123	277.60	1.9669	0.1350

Table S3. SFPs detected as significantly differentially-abundant in response to competition. q-values are calculated by applying a tail-based FDR correction to *p*-values obtained from a linear model iterated over each detected protein. q-values are given both for the effect of mating status (mated/virgin) and the level of competition males were reared in: none (N,1 male), low (L, 2 males), high (H, 8 males). Fold changes are given on a $\log_2$ scale and calculated for each competition comparison within a mating status according to $\chi_{i,j} = \chi_j - \chi_i$ where χ = virgin or mated and *i* and *j* are the first and second competition treatments in the column header, respectively. Functional information associated with each protein's FlyBase entry is provided, along with whether a protein has a known role in sperm competition or in the sex peptide network ('SPNs').

	qval		Virgin fold change			Mated fold change				
Protein	Competition	Mating	N vs H	L vs H	N vs L	N vs H.	L vs H.	N vs L.	Functional class	Function
Acp29AB	0.0280	0.0088	1.0247	0.9525	0.0722	-0.1013	0.1385	-0.2397	C-type lectin	Sperm competition
Acp53C14b	0.0422	0.0129	0.8605	1.3471	-0.4866	0.2556	0.0793	0.1763	Peptide/Prohormone	
antr	0.0030	0.0001	0.5307	0.3408	0.1900	0.1695	0.1584	0.0112	CRISP	SPN
aqrs	0.0350	0.0002	0.5992	0.4379	0.1613	0.2008	0.1301	0.0707	Serine protease	SPN
CG10651	0.0468	0.0139	-0.0270	0.6496	-0.6766	-0.4742	0.1953	-0.6695	CRISP	
CG10730	0.0081	0.0005	0.7607	0.6395	0.1212	0.2794	0.0303	0.2491	Alkaline-phosphatase	
CG11112	0.0015	0.0006	-1.1334	-0.5745	-0.5590	-2.9134	-2.4236	-0.4898	Peptide/Prohormone	
CG11608	0.0091	0.0001	0.6902	0.4270	0.2632	0.5759	0.4127	0.1632	Triglycerol lipase	
CG14034	0.0427	0.0002	0.5093	0.5329	-0.0237	0.2684	0.2113	0.0571	Phospholipase	
CG15116	0.0179	0.5027	0.5075	0.2713	0.2362	0.4173	0.1608	0.2565	Glutathione peroxidase	
CG15117	0.0195	0.0001	0.2742	0.5127	-0.2385	0.3695	0.2043	0.1652	Glycosyl hydrolases	
CG1652	0.0375	0.0002	0.5909	0.4788	0.1120	0.2432	-0.0123	0.2555	C-type lectin	SPN
CG1656	0.0034	0.0011	0.1702	0.4268	-0.2567	0.3956	0.2817	0.1139	C-type lectin	SPN
CG17843	0.0170	0.0001	0.7120	0.6895	0.0225	0.1280	0.0213	0.1067	Thioredoxin	
CG31413	0.0018	0.0001	0.5484	0.6114	-0.0629	0.1038	0.4179	-0.3141		
CG31418	0.0058	0.0001	-0.2645	-0.3228	0.0583	-0.4350	-0.2215	-0.2134	Peptide/Prohormone	
CG31419	0.0468	0.0005	-0.2888	-0.5869	0.2980	0.0245	-0.1950	0.2195		
CG31680	0.0288	0.0001	0.4513	0.1763	0.2750	0.0179	0.2209	-0.2030	Peptide/Prohormone	
CG31684	0.0126	0.0001	0.4958	0.5113	-0.0155	0.1907	0.0400	0.1507		
CG34129	0.0054	0.0001	0.4464	0.2718	0.1745	0.4391	0.2150	0.2241	Serine protease	
CG3640	0.0346	0.0125	0.5862	0.3709	0.2153	0.1954	-0.0176	0.2129	CRISP	
CG43111	0.0046	0.0002	1.1353	0.8502	0.2851	0.1947	0.4254	-0.2307		
CG5162	0.0052	0.0001	0.4184	0.3286	0.0898	0.4096	0.2451	0.1645	Lipase	
CG5267	0.0208	0.1638	-0.4204	-0.4382	0.0178	-0.2502	-0.5942	0.3440	Trypsin protease inhibitor	
CG6071	0.0008	0.0001	0.5891	0.5921	-0.0030	0.3134	0.3984	-0.0851	Metalloprotease	
CG9168	0.0004	0.0726	1.3438	1.3373	0.0065	0.9947	0.5916	0.4031	Phosphatase	
CG9519	0.0123	0.1779	0.7676	1.0329	-0.2652	0.3264	0.4545	-0.1281		
CG9997	0.0027	0.0003	0.3798	0.7352	-0.3553	0.4648	0.4333	0.0315	Serine protease	SPN
Cys	0.0083	0.0083	1.2176	0.5088	0.7088	0.4991	-0.1245	0.6236		
Hexo2	0.0015	0.0001	0.3545	0.1130	0.2416	0.4555	0.1985	0.2570		
intr	0.0009	0.0002	0.5949	0.2968	0.2981	0.4799	0.4434	0.0365		SPN
lectin-29Ca	0.0020	0.0001	0.2151	0.8266	-0.6115	0.3025	0.3679	-0.0655	Lectin	
lectin-30A	0.0487	0.0001	0.1434	0.4122	-0.2688	0.1192	0.1250	-0.0058	Lectin	
mfas	0.0073	0.1730	0.3881	0.2934	0.0948	0.6320	0.1703	0.4617	Fasciclin	
Obp22a	0.0103	0.0001	0.3011	0.5881	-0.2871	-0.1908	0.2383	-0.4291	Oderant binding	
Phm	0.0106	0.0015	0.0138	0.5568	-0.5430	0.1236	0.1958	-0.0722		
regucalcin	0.0016	0.0001	0.5104	0.4427	0.0677	0.3378	0.1837	0.1541		
Sfp24F	0.0103	0.0001	0.4791	0.6907	-0.2116	0.2408	0.2234	0.0173	C-type lectin	
Sfp38D	0.0345	0.0001	1.0045	0.8296	0.1749	0.1022	-0.1665	0.2687		
Sfp51E	0.0484	0.0001	0.6289	0.3316	0.2974	-0.0015	0.1345	-0.1360		
Sfp78E	0.0048	0.0001	-0.1553	-0.2445	0.0892	-0.5872	-0.5704	-0.0168		
Sfp84E	0.0237	0.0172	0.4167	0.1967	0.2200	0.5778	0.5773	0.0004		
Spn28B	0.0057	0.0027	0.6312	0.4895	0.1418	0.5664	0.1483	0.4182	Serpin	
Spn28Db	0.0004	0.0001	-0.4854	0.3506	-0.8361	-1.2096	0.2132	-1.4228		
Spn75F	0.0031	0.0001	0.4453	0.4879	-0.0427	0.1969	0.1421	0.0548	Serpin	

(continued)

Table S4. No significant association between differentially-abundant SFPs and miRNA regulators. Effects of measured variables from a linear mixed effects analysis on the abundance of detected SFPs for which a putative miRNA regulator has been proposed (see main text). Included variables were the mating status of males in the sample (mated or virgin), the competition group they were reared in (none, 1 male; low, 2 males; high, 8 males), the proposed miRNA regulator, and the experimental replicate. Significant *p*-values at the <0.05 levels are given in red. Corresponding data are plotted in Figure S2.

Effect	*NumDf*	*Sum of sq*	*Mean sq*	*F*	*P*
*Competition*Mating*miRNA*	6	1.0595	0.1766	0.5993	0.7312
*Competition*miRNA*	6	0.6227	0.1038	0.3528	0.9085
*Competition*Mating*	2	9.0465	9.0465	30.8211	<0.0001
*Mating*miRNA*	3	0.6796	0.6796	2.3171	0.0740
miRNA	3	0.0023	0.0023	0.0077	0.9991
Replicate	4	3.8397	3.8397	13.0818	<0.0001

Table S5. Elevated mating duration in response to the presence of competition is lost after two matings. Males were held alone (no competition), in a single-sex pair (low competition), or in a single-sex group of 8 (high competition) before being provided with 5 successive virgin females. (*A*) Contribution and significance of fixed effects in a linear mixed effects model fitted to mating duration data. Male identity was included as a random effect. (*B*) Pairwise comparisons between each group size treatment within a particular mating. Significant values at the *p*<0.05 level are given in red.

A

Effect	*NumDf*	*Sum of sq*	*Mean sq*	*F*	*P*
*Competition*Mating number*	8	161.3	20.15	2.03	0.0400
Competition	2	129.8	64.91	6.50	0.0017
Mating number	4	4417.2	1104.31	110.51	<0.0001
Replicate	1	25.4	25.35	2.54	0.1121

B

Competition	*Mating*	*Estimate*	*SE*	*Df*	*t.ratio*	*p.value*
**None - Low**	1	-1.7447	0.4500	1491	-3.877	0.0001
**None - High**	1	-1.6816	0.4769	1491	-3.526	0.0004
**Low - High**	1	0.0631	0.4654	1490	0.136	0.8922
**None - Low**	2	-1.4633	0.4500	1491	-3.252	0.0012
**None - High**	2	-1.0062	0.4769	1491	-2.110	0.0350
**Low - High**	2	0.4571	0.4654	1490	0.982	0.3262
**None - Low**	3	-0.2153	0.4500	1491	-0.478	0.6324
**None - High**	3	-0.5387	0.4769	1491	-1.130	0.2588
**Low - High**	3	-0.3234	0.4653	1490	-0.695	0.4872
**None - Low**	4	-0.8684	0.4500	1491	-1.930	0.0538
**None - High**	4	-0.5158	0.4769	1491	-1.082	0.2796
**Low - High**	4	0.3526	0.4653	1490	0.758	0.4488
**None - Low**	5	0.0436	0.4500	1491	0.097	0.9228
**None - High**	5	-0.4250	0.4769	1491	-0.891	0.3730
**Low - High**	5	-0.4686	0.4653	1490	-1.007	0.3141
